# Supplementary material for: Metagenome diversity illuminates the origins of pathogen effectors
Source: mBio. 2024 Apr 2;15(5):e00759-23. doi: 10.1128/mbio.00759-23 (PMC11077975; doi:10.1128/mbio.00759-23)
Supplement: Fig. S1-S5 — Supplemental figures. [file mbio.00759-23-s0001.pdf]

FIGURE S1. Characteristics of the Rickettsiales *vir* homolog (*rvh*) type IV secretion system (T4SS). (A) Schema depicting genome-based Rickettsiales phylogeny estimation, after Schön *et al.* (28). Host dependency evolved after the divergence of basal families Mitibacteraceae and Athabascaceae (orange). The *rvh* T4SS was present in the Rickettsiales ancestor (yellow). **(B)** Description of the general *rvh* T4SS characteristics, summarized from prior studies (38, 42, 45, 89). **(C)** *rvh* genes are arrayed in clustered islets or single transcriptional units throughout *Rickettsia* genomes (e.g., *R. typhi* str. Wilmington). Asterisks denote genes for RvhB9-II, RvhB8-II and RvhB4-II. Similar *rvh* gene arrangements are found in other Rickettsiales genomes (38). **(D)** Proposed model for conserved *rvh* gene duplication (*rvhB9*, *rvhB8* and *rvhB4*) based on divergent characteristics of RvhB9-II, RvhB8-II and RvhB4-II (44, 45).

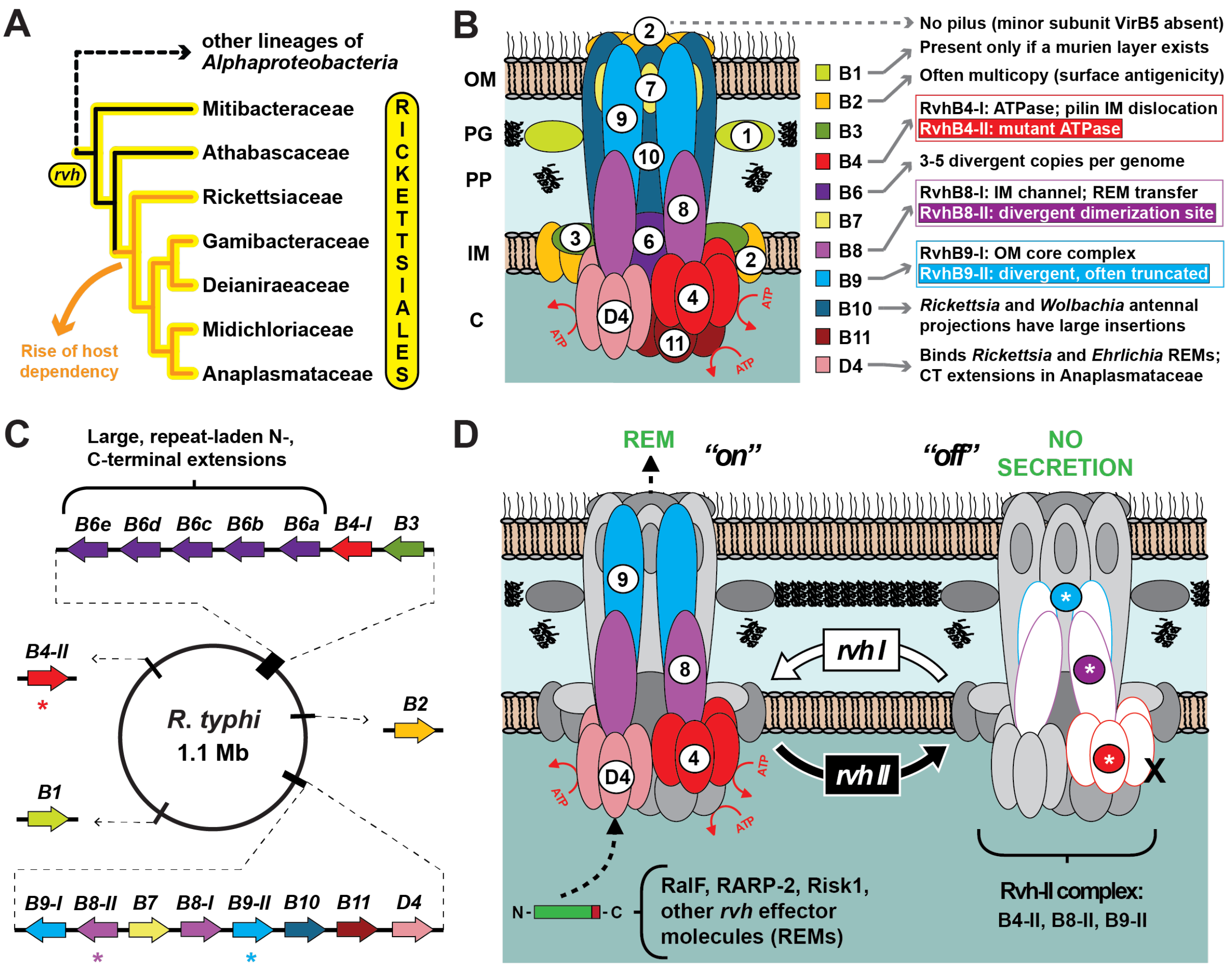
A
other lineages of
Alphaproteobacteria
Mitibacteraceae
Athabascaceae
Rickettsiaceae
Gamibacteraceae
Deianiraeaceae
Midichloriaceae
Anaplasmataceae
RICKETTSIALES
rvh
Rise of host dependency
B
OM
PG
PP
IM
C
B1
B2
B3
B4
B6
B7
B8
B9
B10
B11
D4
No pilus (minor subunit VirB5 absent)
Present only if a murien layer exists
Often multicopy (surface antigenicity)
RvhB4-I: ATPase; pilin IM dislocation
RvhB4-II: mutant ATPase
3-5 divergent copies per genome
RvhB8-I: IM channel; REM transfer
RvhB8-II: divergent dimerization site
RvhB9-I: OM core complex
RvhB9-II: divergent, often truncated
Rickettsia and Wolbachia antennal projections have large insertions
Binds Rickettsia and Ehrlichia REMs; CT extensions in Anaplasmataceae
ATP
C
Large, repeat-laden N-, C-terminal extensions
B6e B6d B6c B6b B6a B4-I B3
B4-II
B2
B1
R. typhi
1.1 Mb
B9-I B8-II B7 B8-I B9-II B10 B11 D4
D
REM
"on"
"off"
NO SECRETION
rvh I
rvh II
D4
N- -C
RalF, RARP-2, Risk1, other rvh effector molecules (REMs)
Rvh-II complex:
B4-II, B8-II, B9-II

Fig. S1

FIGURE S2. RvhB4-based phylogeny estimation. Phylogeny estimated from concatenated alignments of RvhB4-I and RvhB4-II proteins from 153 rickettsial genome assemblies. *Agrobacterium tumefaciens* str. F4 VirB4 was used as an outgroup. See **Table S1** for sequence information. RvhB4-I and RvhB4-II proteins were separately aligned using MUSCLE (default parameters) with both alignments concatenated (1974 positions). TRIMAL (184) was used to create a second dataset with less conserved positions masked (1613 positions). For both unmasked and masked alignments, a maximum likelihood-based phylogeny was estimated with PhyML (185), using the Smart Model Selection (186) tool to determine the best substitution matrix and model for rates across aa sites (LG (G+I+F) for both alignments). Branch support was assessed with 1,000 pseudo-replications. **(A)** Statistics for the phylogeny estimated on the unmasked alignment, which is shown as a phylogram in panels **C** and **D**, and as a cladogram in **FIG. 1** and **FIG. 2**. **(B)** Statistics for the phylogeny estimated on the masked alignment. This tree is congruent in topology and branch support to the tree generated on the unmasked alignment. **(C)** Non-Rickettsiaceae lineages (taxa not previously described at the family level are in black text). **(D)** Rickettsiaceae lineages. "*Candidatus* Sneabacter namystus" (arrow), which lacks *rvh* genes but carries a type VI secretion system (see **FIG. S3**) is shown on the phylogram per prior phylogeny estimation (187, 188). Black boxes provide short names for 29 MAGs from Davison *et al*. (73) (NOTE: the green colored clade comprises genus *Tisiphia* though "*Rickettsia*" reflects NCBI taxonomy as of Feb. 26th, 2023). Asterisks, multiple genome assemblies for a species. STG, Scrub Typhus Group; BG, Bellii Group; TRG, Transitional Group; TG, Typhus Group; TIG, Tamurae-Ixodes Group; SFG, Spotted Fever Group.

Fig. S2

A **PhyML statistics (unmasked):**

. Model of aa substitution:	LG (+G+I+F)
. Number of taxa:	153
. Log-likelihood:	-177643.39527
. Unconstrained log-likelihood:	-14907.80653
. Composite log-likelihood:	-866892.01804
. Tree size:	52.48531
. Discrete gamma model:	Yes
- Number of classes:	4
- Gamma shape parameter:	0.883
- Relative rate in class 1:	0.11306 [freq=0.250000]
- Relative rate in class 2:	0.43852 [freq=0.250000]
- Relative rate in class 3:	0.97754 [freq=0.250000]
- Relative rate in class 4:	2.47088 [freq=0.250000]
. Proportion of invariant:	0.039

B **PhyML statistics (masked):**

. Model of aa substitution:	LG (+G+I+F)
. Number of taxa:	153
. Log-likelihood:	-170882.36583
. Unconstrained log-likelihood:	-11869.35617
. Composite log-likelihood:	-708273.33174
. Tree size:	48.13634
. Discrete gamma model:	Yes
- Number of classes:	4
- Gamma shape parameter:	0.891
- Relative rate in class 1:	0.11473 [freq=0.250000]
- Relative rate in class 2:	0.44135 [freq=0.250000]
- Relative rate in class 3:	0.97930 [freq=0.250000]
- Relative rate in class 4:	2.46463 [freq=0.250000]
. Proportion of invariant:	0.045

C

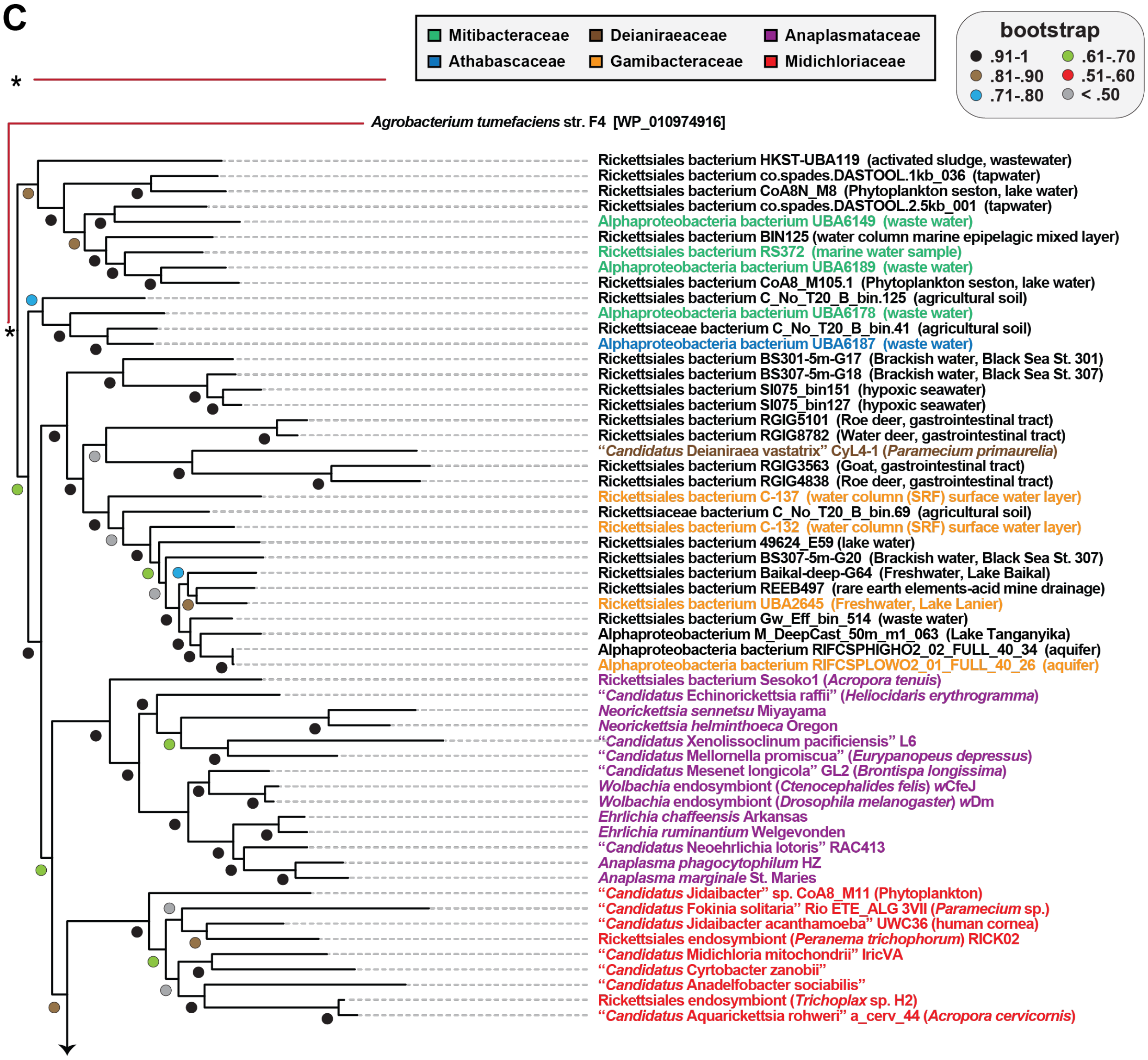

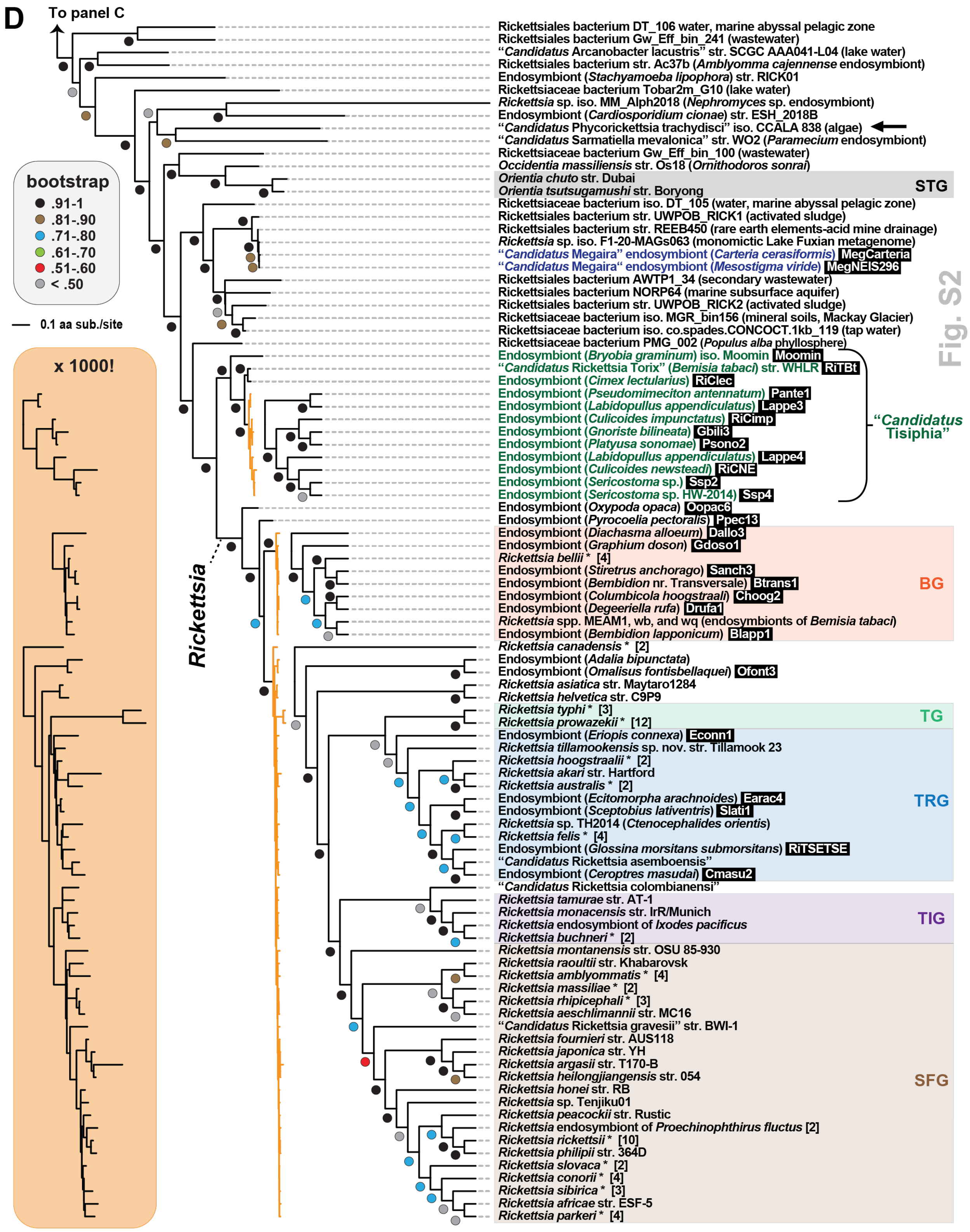
D
To panel C
bootstrap
.91-1
.81-.90
.71-.80
.61-.70
.51-.60
< .50
0.1 aa sub./site
x 1000!
Rickettsia
Rickettsiales bacterium DT_106 water, marine abyssal pelagic zone
Rickettsiales bacterium Gw_Eff_bin_241 (wastewater)
"Candidatus Arcanobacter lacustris" str. SCGC AAA041-L04 (lake water)
Rickettsiales bacterium str. Ac37b (Amblyomma cajennense endosymbiont)
Endosymbiont (Stachyamoeba lipophora) str. RICK01
Rickettsiaceae bacterium Tobar2m_G10 (lake water)
Rickettsia sp. iso. MM_Alph2018 (Nephromyces sp. endosymbiont)
Endosymbiont (Cardiosporidium cionae) str. ESH_2018B
"Candidatus Phycorickettsia trachydisci" iso. CCALA 838 (algae)
"Candidatus Sarmatiella mevalonica" str. WO2 (Paramecium endosymbiont)
Rickettsiaceae bacterium Gw_Eff_bin_100 (wastewater)
Occidentia massiliensis str. Os18 (Ornithodoros sonrai)
Orientia chuto str. Dubai
Orientia tsutsugamushi str. Boryong
STG
Rickettsiaceae bacterium iso. DT_105 (water, marine abyssal pelagic zone)
Rickettsiales bacterium str. UWPOB_RICK1 (activated sludge)
Rickettsiales bacterium str. REEB450 (rare earth elements-acid mine drainage)
Rickettsia sp. iso. F1-20-MAGs063 (monomictic Lake Fuxian metagenome)
"Candidatus Megaira" endosymbiont (Carteria cerasiformis) MegCarteria
"Candidatus Megaira" endosymbiont (Mesostigma viride) MegNEIS296
Rickettsiales bacterium AWTP1_34 (secondary wastewater)
Rickettsiales bacterium NORP64 (marine subsurface aquifer)
Rickettsiales bacterium str. UWPOB_RICK2 (activated sludge)
Rickettsiaceae bacterium iso. MGR_bin156 (mineral soils, Mackay Glacier)
Rickettsiaceae bacterium iso. co.spades.CONCOCT.1kb_119 (tap water)
Rickettsiaceae bacterium PMG_002 (Populus alba phyllosphere)
Endosymbiont (Bryobia graminum) iso. Moomin Moomin
"Candidatus Rickettsia Torix" (Bemisia tabaci) str. WHLR RiTBt
Endosymbiont (Cimex lectularius) RiClec
Endosymbiont (Pseudomimeciton antennatum) Pante1
Endosymbiont (Labidopullus appendiculatus) Lappe3
Endosymbiont (Culicoides impunctatus) RiCimp
Endosymbiont (Gnoriste bilineata) Gbili3
Endosymbiont (Platyusa sonomae) Psono2
Endosymbiont (Labidopullus appendiculatus) Lappe4
Endosymbiont (Culicoides newsteadi) RiCNE
Endosymbiont (Sericostoma sp.) Ssp2
Endosymbiont (Sericostoma sp. HW-2014) Ssp4
"Candidatus Tisiphia"
Endosymbiont (Oxypoda opaca) Oopac6
Endosymbiont (Pyrocoelia pectoralis) Ppec13
Endosymbiont (Diachasma alloeum) Dallo3
Endosymbiont (Graphium doson) Gdoso1
Rickettsia bellii * [4]
Endosymbiont (Stiretrus anchorago) Sanch3
Endosymbiont (Bembidion nr. Transversale) Btrans1
Endosymbiont (Columbicola hoogstraali) Choog2
Endosymbiont (Degeeriella rufa) Drufa1
Rickettsia spp. MEAM1, wb, and wq (endosymbionts of Bemisia tabaci)
Endosymbiont (Bembidion lapponicum) Blapp1
BG
Rickettsia canadensis * [2]
Endosymbiont (Adalia bipunctata)
Endosymbiont (Omalisus fontisbellaquei) Ofont3
Rickettsia asiatica str. Maytaro1284
Rickettsia helvetica str. C9P9
Rickettsia typhi * [3]
Rickettsia prowazekii * [12]
TG
Endosymbiont (Eriopis connexa) Econn1
Rickettsia tillamookensis sp. nov. str. Tillamook 23
Rickettsia hoogstraalii * [2]
Rickettsia akari str. Hartford
Rickettsia australis * [2]
Endosymbiont (Ecitomorpha arachnoides) Earac4
Endosymbiont (Sceptobius lativentris) Slati1
Rickettsia sp. TH2014 (Ctenocephalides orientis)
Rickettsia felis * [4]
Endosymbiont (Glossina morsitans submorsitans) RiTSETSE
"Candidatus Rickettsia asemboensis"
Endosymbiont (Ceroptres masudai) Cmasu2
TRG
"Candidatus Rickettsia colombianensi"
Rickettsia tamurae str. AT-1
Rickettsia monacensis str. IrR/Munich
Rickettsia endosymbiont of Ixodes pacificus
Rickettsia buchneri * [2]
TIG
Rickettsia montanensis str. OSU 85-930
Rickettsia raoultii str. Khabarovsk
Rickettsia amblyommatis * [4]
Rickettsia massiliae * [2]
Rickettsia rhipicephali * [3]
Rickettsia aeschlimannii str. MC16
"Candidatus Rickettsia gravesii" str. BWI-1
Rickettsia fournieri str. AUS118
Rickettsia japonica str. YH
Rickettsia argasii str. T170-B
Rickettsia heilongjiangensis str. 054
Rickettsia honei str. RB
Rickettsia sp. Tenjiku01
Rickettsia peacockii str. Rustic
Rickettsia endosymbiont of Proechinophthirus fluctus [2]
Rickettsia rickettsii * [10]
Rickettsia philipii str. 364D
Rickettsia slovaca * [2]
Rickettsia conorii * [4]
Rickettsia sibirica * [3]
Rickettsia africae str. ESF-5
Rickettsia parkeri * [4]
SFG
Fig. S2

FIGURE S3. Non-orthologous replacement of secretion machines in Rickettsiales. (A) Predicted proteins encoded on the "*Candidatus* Sneabacter namystus" unnamed plasmid, which mostly encodes type VI secretion system (T6SS) components. Protein colors depict their position in panel **B**. Plasmid map created with Proksee (https://proksee.ca/). **(B)** Model of a typical T6SS. The assignment of QEK39893 as a TssD protein is putative (see panel **D**). **(C)** HaloBlast analysis of 12 "*Candidatus* S. namystus" T6SS proteins. BlastP searches were performed against specified NCBI taxon databases (see **TABLE S3** for retrieved sequences). Highlighting: *Sm* score (= *b* * *I* * *Q*, where *b* is the bitscore of the match, *I* is the percent identity, and *Q* is the percent length of the query that aligned) (26, 81, 204). **(D)** Alignment of the putative TssD protein of "*Candidatus* S. namystus" with annotated TssD proteins from *Gemmata palustris* (Planctomycetia, WP_210656154) and *Denitrobaculum tricleocarpae* (Rhodospirillales, WP_142896164). Alignment done with MUSCLE (189) using default parameters. Amino acid coloring is described in the **FIG. 3** legend. **(E)** Detection of a second complete T6SS in the MAG Rickettsiales endosymbiont NP11 (NCBI BioSample SAMN07620291) (205). Another MAG, Rickettsiales endosymbiont EAC13 (SAMN07620031) (205), contained fewer T6SS genes. Neither MAG harbors *rvh* genes (data not shown), indicating that other Rickettsiales species may utilize a T6SS in place of a T4SS.

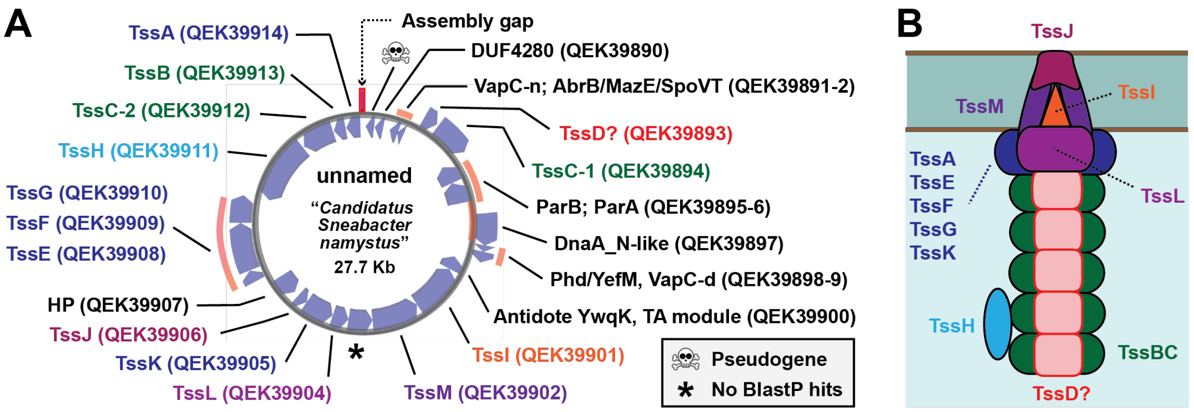

C

Taxon database	TssC-1 (QEK39894) Subj.	x̄ *Sm*	top 5 *Sm*	TssI (QEK39901) Subj.	x̄ *Sm*	top 5 *Sm*	TssM (QEK39902) Subj.	x̄ *Sm*	top 5 *Sm*
Rickettsiaceae, "rick"	1	3938.92	na	0	na	na	0	na	na
Rickettsiales (-Rickettsiaceae), "Rick"	5	3110.16	3110.16	0	na	na	0	na	na
Alphaproteobacteria (- Rickettsiales), "Alph"	100	**4291.79**	4937.88	0	na	na	100	1277.14	1623.35
Proteobacteria (- Bacteria), "Prot"	100	4277.22	5023.09	0	na	na	100	**1540.32**	**1936.53**
Bacteria (- *Proteobacteria*), "Bact"	100	4128.62	**5128.33**	9	**235.22**	**248.28**	10	863.72	1208.91
Top hit:	*Denitrobaculum tricleocarpae*			*Bacteroides fragilis*			*Gallaecimonas pentaromativorans*		

	TssL (QEK39904) Subj.	x̄ *Sm*	top 5 *Sm*	TssK (QEK39905) Subj.	x̄ *Sm*	top 5 *Sm*	TssE (QEK39908) Subj.	x̄ *Sm*	top 5 *Sm*	TssF (QEK39909) Subj.	x̄ *Sm*	top 5 *Sm*	TssG (QEK39910) Subj.	x̄ *Sm*	top 5 *Sm*
"rick"	0	na	na	0	na	na	0	na	na	0	na	na	0	na	na
"Rick"	1	1356.85	na	1	1304.62	na	0	na	na	2	810.13	na	1	1441.62	na
"Alph"	100	1082.10	1427.30	100	3490.63	3882.21	100	928.76	1266.74	100	3127.41	3636.80	100	**1587.68**	**2182.89**
"Prot"	100	**1505.68**	**1595.68**	100	**4011.95**	**4577.02**	100	**1186.43**	1600.91	100	**3407.50**	**4185.17**	100	1579.76	1896.50
"Bact"	59	902.58	1258.88	100	2862.49	3642.10	100	1163.66	**1645.97**	100	3124.52	3734.46	100	1433.84	1809.90
Top hit:	*Pseudomonas* sp. NFACC02			*Proteobacteria* bacterium SZAS-81			*Gammaproteobacteria* bacterium co.spades.METABAT.1kb_077			*Aliikangiella coralliicola*			*Roseibium aggregatum*		

	TssH (QEK39911) Subj.	x̄ *Sm*	top 5 *Sm*	TssC-2 (QEK39912) Subj.	x̄ *Sm*	top 5 *Sm*	TssB (QEK39913) Subj.	x̄ *Sm*	top 5 *Sm*	TssA (QEK39914) Subj.	x̄ *Sm*	top 5 *Sm*
"rick"	100	9459.90	10581.94	1	3561.79	na	0	na	na	0	na	na
"Rick"	100	10694.34	13281.06	5	20284.61	20284.61	3	5591.07	5591.07	1	584.94	na
"Alph"	100	19031.95	20984.22	100	34628.35	36706.21	100	**9200.88**	10411.30	100	453.61	668.13
"Prot"	100	18957.91	**22359.96**	100	**36188.30**	37845.80	100	8628.27	10178.60	100	**732.63**	1013.44
"Bact"	100	**19737.08**	21359.54	100	34537.02	**39342.97**	100	8864.51	**10427.33**	100	622.01	**1200.14**
Top hit:	unclassified *Acinetobacter*			*Gemmata palustris*			*Gammaproteobacteria* bacterium HyVt-542			*Rubinisphaera* sp.		

Fig. S3

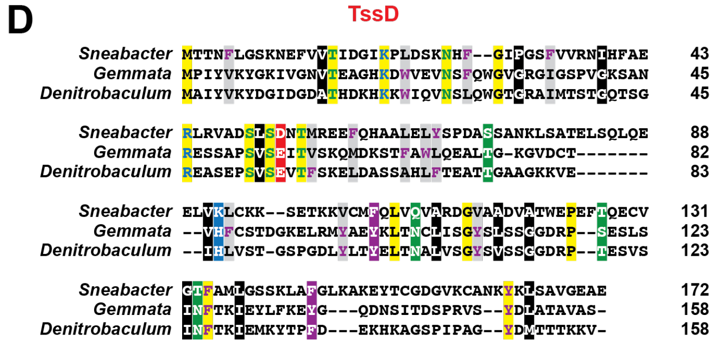

E

Rickettsiales bacterium NP11

"*Candidatus* Sneabacter namystus"

Protein	NCBI acc. no.	%ID
TssD	MBV34071	17.12
TssC-1	MBV34078	26.17
TssI	ns	ns
TssM	MBV34981	19.86
TssL	MBV34982	28.57
TssK	MBV34983	22.01
TssE	MBV34075	22.07
TssF	MBV34074	24.65
TssG	MBV34073	26.67
TssH	MBV34072	42.34
TssC-2	MBV34976	60.04
TssB	MBV34977	54.27
TssA	MBV34978	33.33

FIGURE S4. Phylogenomics and bioinformatics analyses of RalF proteins. Amino acid coloring is described in the FIG. 3 legend. Black boxes provide short names for MAGs from Davison *et al*. (73). These and additional newly discovered RalF-like proteins (highlighted yellow) substantially expand the prior RalF diversity. **(A, B)** Sequence logos (190) illustrating conservation within two novel domains present in some *Legionella* and *Rickettsia* proteins. Sequence information is provided in **Table S2**. Sequences were aligned with MUSCLE (189) using default parameters. These two domains are carried in a single protein (MCC8377991) from *Rickettsia* endosymbiont of *Graphium doson* (Gdoso1). **(A)** Sequence conservation (*n* = 18) within the Gdoso1 MCC8377991 central domain. **(B)** Sequence conservation (*n* = 56) within the Gdoso1 MCC8377991 C-terminal domain. **(C)** Structural characteristics of the RalF S7D-SCD architecture. ***Left***: *L. pneumophila* RalF structure (PDB 4C7P) (86). The delineation of the Sec7 domain (S7D, red) and Sec7-capping domain (SCD, green) is shown, with an approximation of the active site Glu (asterisk). ***Right***: regions of variability within the predicted structures of *R. typhi* str. Wilmington (RT0362) and *R. bellii* str. RML369-C (RBE_0868) RalF proteins. Modeling done with Phyre2 (195). *Rickettsia* full length RalF proteins contain an extended C-terminal domain relative to *L. pneumophila* RalF (56). **(D-F)** Gallery of predicted structures for diverse RalF and RalF-like proteins. All protein domains are described in the gray inset. **(G)** Sequence comparison of diverse RalF and RalF-like proteins (S7D and SCD only). An initial alignment with MUSCLE (default parameters) was manually adjusted with reference to the Phyre2 structure models to *L. pneumophila* RalF (PDB 4C7P). The secondary structure of *L. pneumophila* RalF is superimposed over the alignment. Conserved residues are highlighted yellow. S7D: two

highly conserved regions (motif 1 and motif 2) that together form the Sec7 active site are boxed in red (206); the active site Glu of Motif 1, which is essential for Arf1 recruitment to the LCV (85) and Arf6 recruitment to the plasma membrane during *Rickettsia* invasion (148), is noted with an asterisk; divergent residues within motif 2 of *R*. *bellii* RalF are colored black. SCD: aromatic clusters comprising the membrane sensor region are enclosed in purple boxes; following previous mutagenesis analysis of the SCD (87), # denotes residues in *L*. *pneumophila* RalF permuted to the corresponding residues in *R*. *prowazekii* RalF (and vice versa) and ^ denotes residues in *L*. *pneumophila* RalF permuted to the corresponding residues in *R*. *prowazekii* RalF but not reciprocated. The conserved KATY motif, which contacts the S7D remote from the active site and is thought to function as a hinge for the conformational change that activates RalF (87), is colored black.

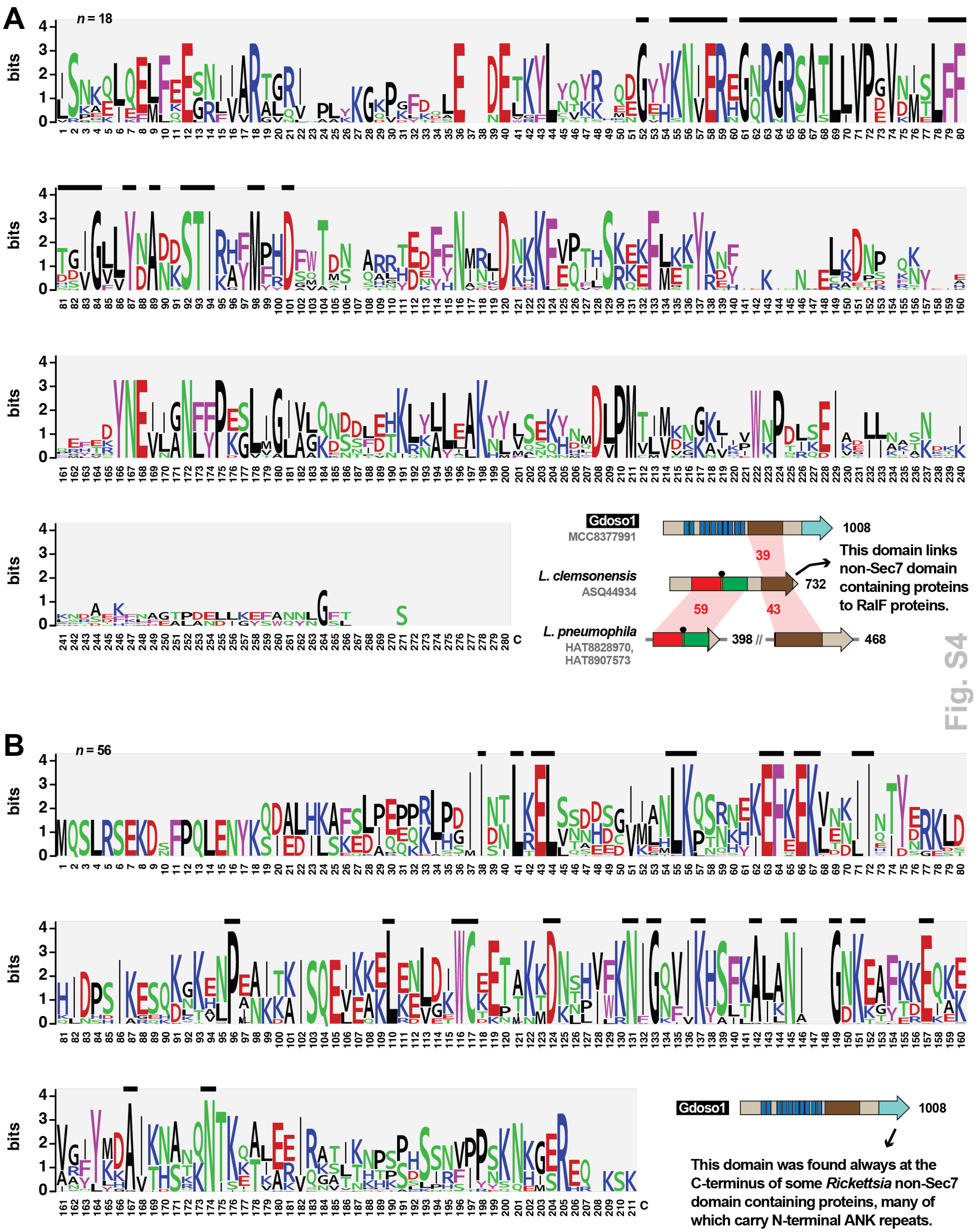

A
n = 18
bits
Gdoso1
MCC8377991
1008
39
This domain links non-Sec7 domain containing proteins to RalF proteins.
L. clemsonensis
ASQ44934
732
59
43
L. pneumophila
HAT8828970, HAT8907573
398 //
468
Fig. S4
B
n = 56
bits
Gdoso1
1008
This domain was found always at the C-terminus of some Rickettsia non-Sec7 domain containing proteins, many of which carry N-terminal ANK repeats.

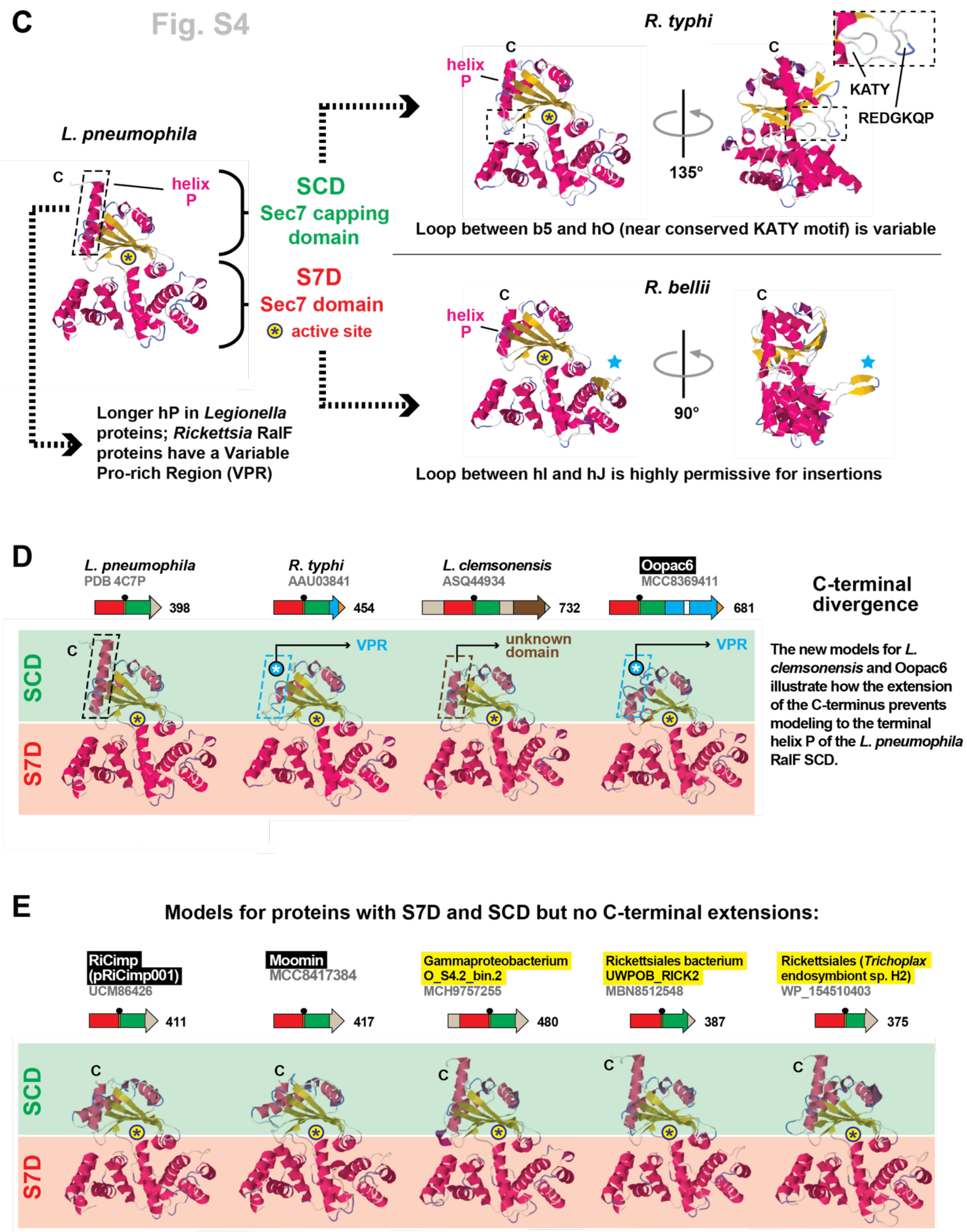
C
Fig. S4
L. pneumophila
C
helix P
SCD
Sec7 capping domain
S7D
Sec7 domain
active site
Longer hP in Legionella proteins; Rickettsia RalF proteins have a Variable Pro-rich Region (VPR)
R. typhi
C
helix P
135°
KATY
REDGKQP
Loop between b5 and hO (near conserved KATY motif) is variable
R. bellii
C
helix P
90°
Loop between hI and hJ is highly permissive for insertions
D
L. pneumophila
PDB 4C7P
398
R. typhi
AAU03841
454
L. clemsonensis
ASQ44934
732
Oopac6
MCC8369411
681
C-terminal divergence
SCD
S7D
C
VPR
unknown domain
VPR
The new models for L. clemsonensis and Oopac6 illustrate how the extension of the C-terminus prevents modeling to the terminal helix P of the L. pneumophila RalF SCD.
E
Models for proteins with S7D and SCD but no C-terminal extensions:
RiCimp (pRiCimp001)
UCM86426
411
Moomin
MCC8417384
417
Gammaproteobacterium O_S4.2_bin.2
MCH9757255
480
Rickettsiales bacterium UWPOB_RICK2
MBN8512548
387
Rickettsiales (Trichoplax endosymbiont sp. H2)
WP_154510403
375
SCD
S7D
C

Fig. S4

F **Models for proteins with no (or degraded) S7D; N- or C-terminal extensions may be present**

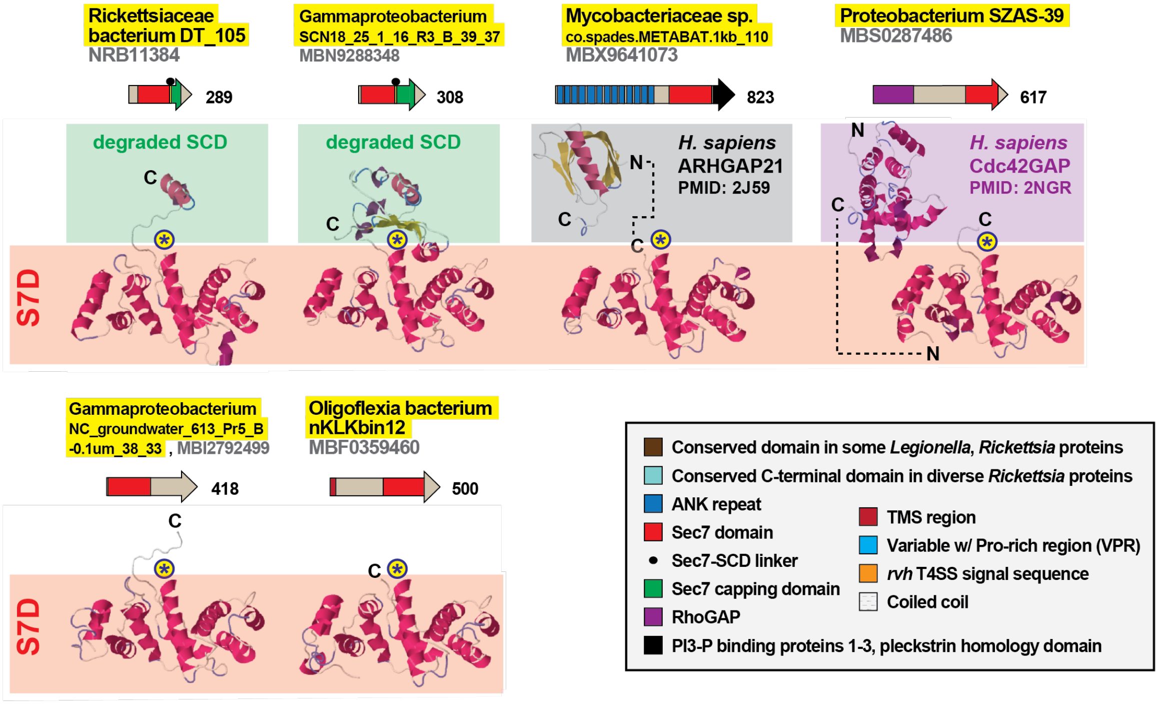

G

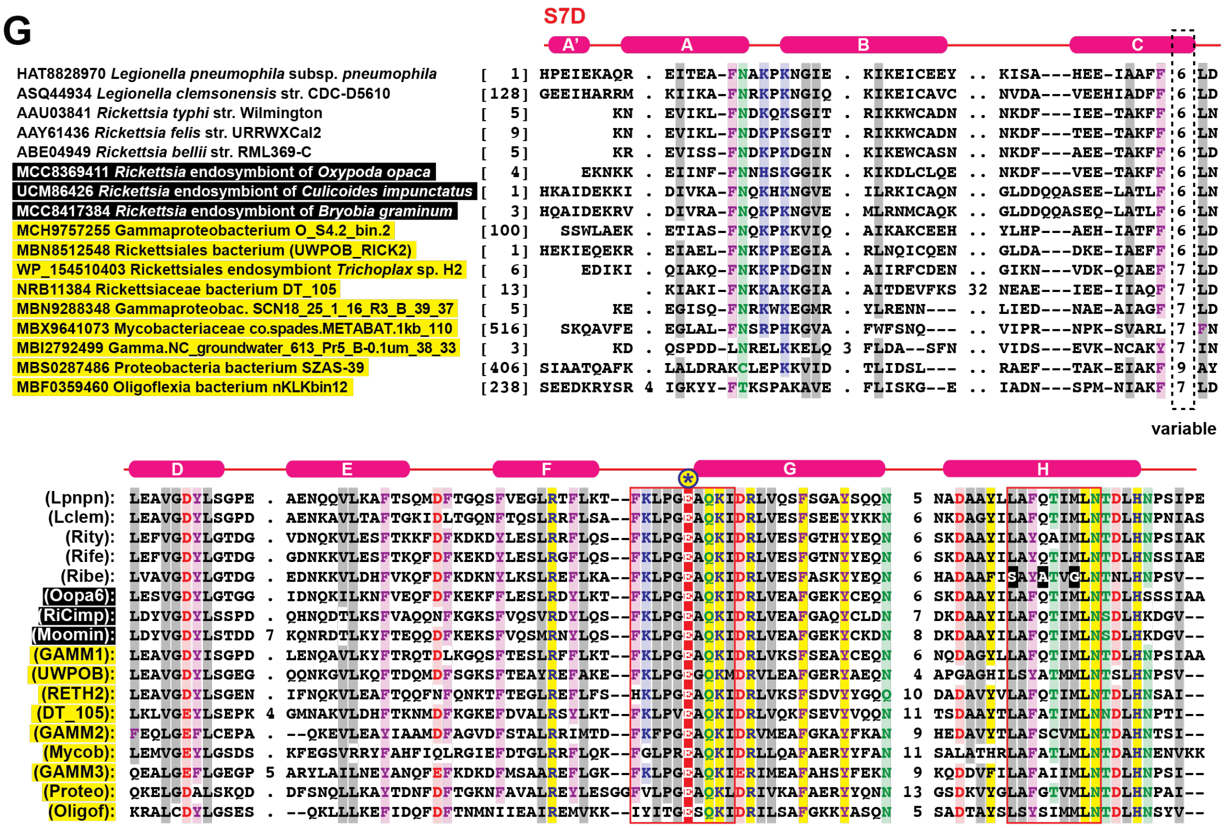

G (cont'd.)

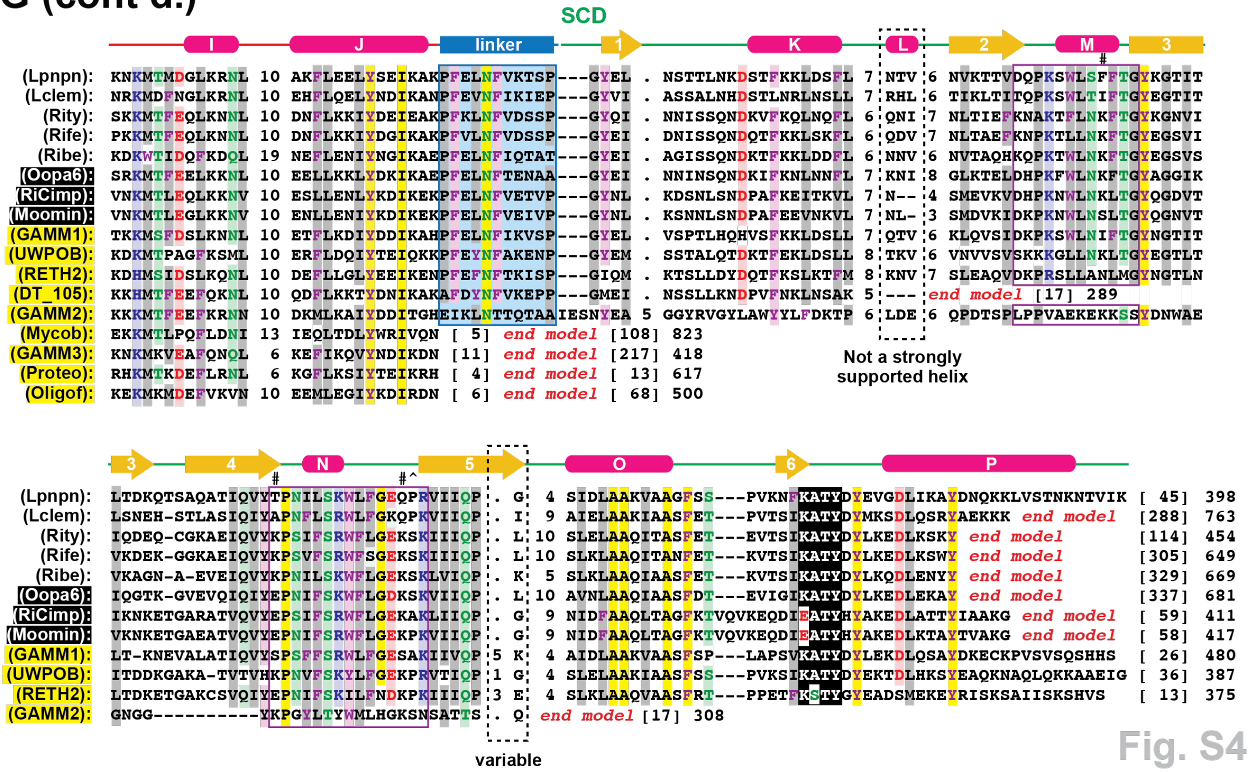

Fig. S4

FIGURE S5. The N-terminal domain of *Rickettsia* Sca4 proteins is recurrent and widespread in other rickettsial proteins. Amino acid coloring is described in the FIG. 3 legend. Black boxes provide short names for MAGs from Davison *et al*. (73). **(A)** Alignment of the Schuenke Walker Antigen (SWA) domains of select *Rickettsia* Sca4 proteins with analogous domains in SWA-Risk2 chimeras (SWA-PIK) and SWA modular proteins (SWAMPs). Domains were retrieved from BlastP searches against the NCBI nr protein database using the *R. typhi* SWA domain as a query. Sequences were aligned with MUSCLE (189) using default parameters. Structural assignment above alignment corresponds to the modeling of *Rickettsia* Sca4 N-terminal domains to the *R. rickettsii* SWA domain (PDB ID: 4LQ8) (112). **(B)** Illustration of the SWA domain of *R. typhi* Sca4 and the two Vinculin Binding Sites (VBS); VBS-C of *R. rickettsii* interacts with the head domain of human vinculin (76). **(C)** Gallery of diverse architectures for select SWAMPs and predicted structures of SWA domains. Phyre2 (195) was used to model all SWA structures to the *R. rickettsii* SWA domain, as well as the PIK domain of Rickettsiales bacterium str. UWPOB_RICK1 to a portion of the LepB protein of *L. pneumophila* (PDB ID: 4JW1) (201). Domains on schemas were predicted with SMART (191). **(D)** Sequence logos (190) depict a consensus from an alignment of all 385 SWA domains retrieved from a BlastP searches against the NCBI nr protein database using the *R. typhi* SWA domain as a query (sequence information in Table S2).

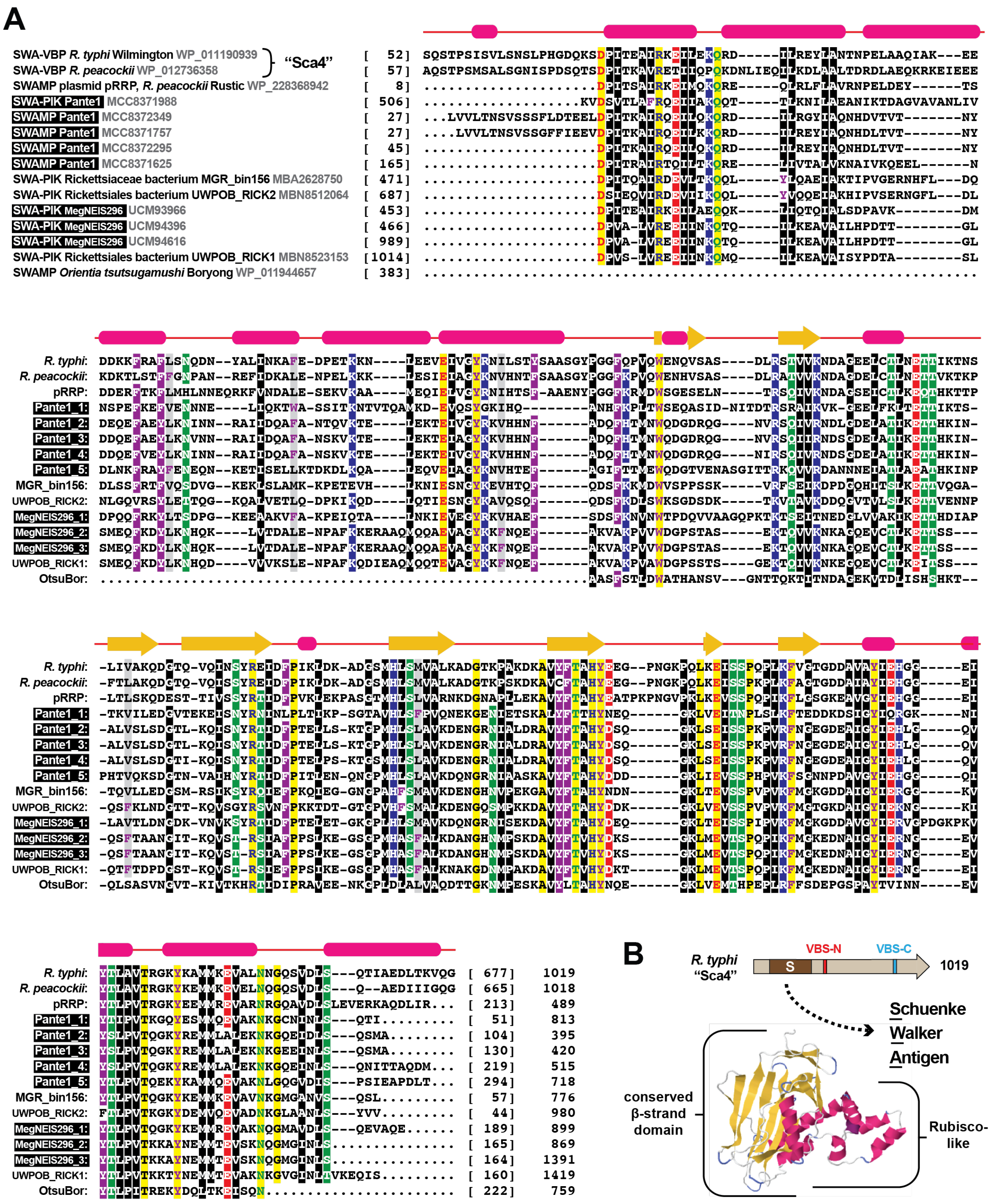
A
SWA-VBP R. typhi Wilmington WP_011190939
SWA-VBP R. peacockii WP_012736358
"Sca4"
SWAMP plasmid pRRP, R. peacockii Rustic WP_228368942
SWA-PIK Pante1 MCC8371988
SWAMP Pante1 MCC8372349
SWAMP Pante1 MCC8371757
SWAMP Pante1 MCC8372295
SWAMP Pante1 MCC8371625
SWA-PIK Rickettsiaceae bacterium MGR_bin156 MBA2628750
SWA-PIK Rickettsiales bacterium UWPOB_RICK2 MBN8512064
SWA-PIK MegNEIS296 UCM93966
SWA-PIK MegNEIS296 UCM94396
SWA-PIK MegNEIS296 UCM94616
SWA-PIK Rickettsiales bacterium UWPOB_RICK1 MBN8523153
SWAMP Orientia tsutsugamushi Boryong WP_011944657
R. typhi:
R. peacockii:
pRRP:
Pante1_1:
Pante1_2:
Pante1_3:
Pante1_4:
Pante1_5:
MGR_bin156:
UWPOB_RICK2:
MegNEIS296_1:
MegNEIS296_2:
MegNEIS296_3:
UWPOB_RICK1:
OtsuBor:
B
R. typhi
"Sca4"
S
VBS-N
VBS-C
1019
Schuenke
Walker
Antigen
conserved
β-strand
domain
Rubisco-
like

Fig. S5

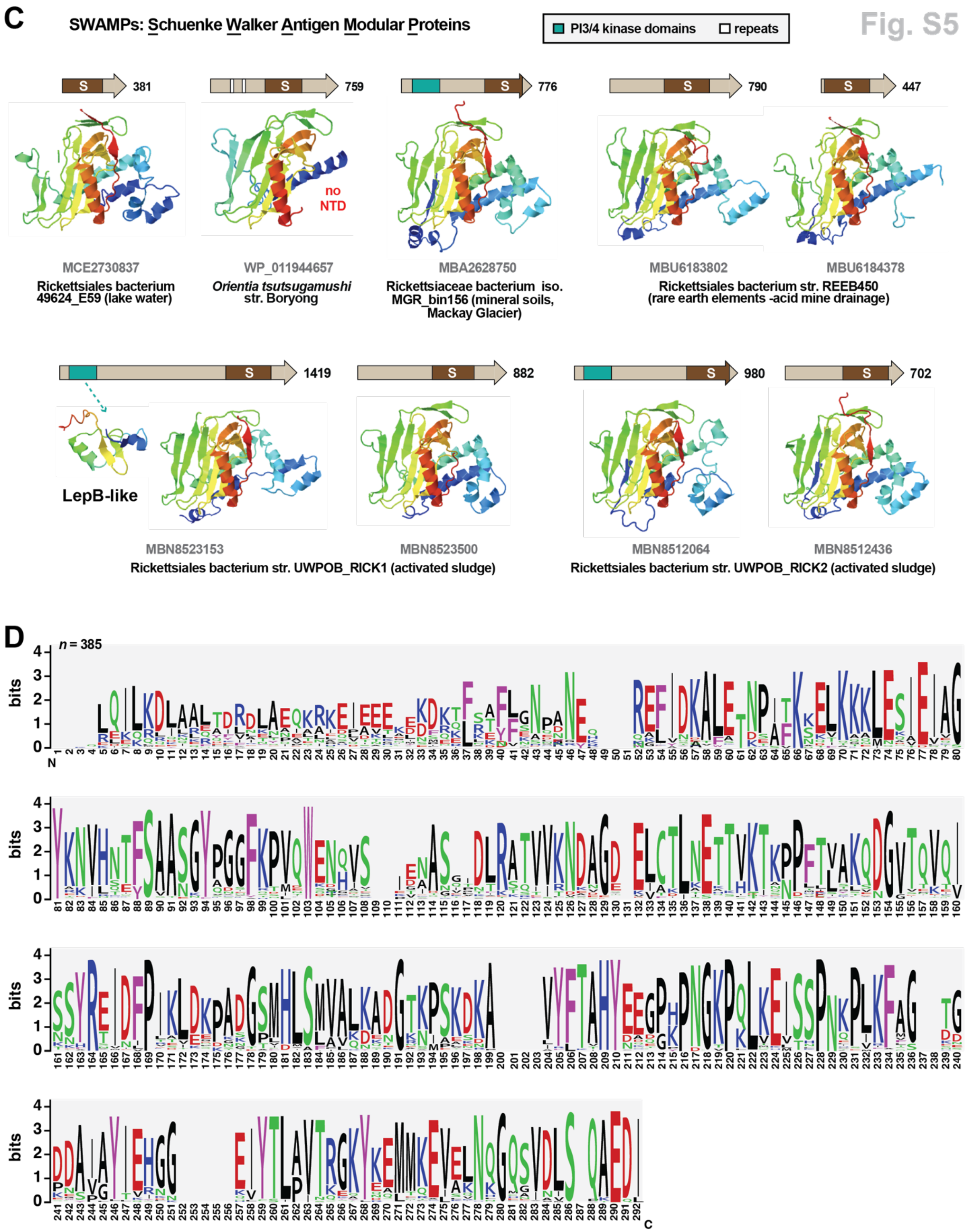
C
SWAMPs: Schuenke Walker Antigen Modular Proteins
PI3/4 kinase domains
repeats
Fig. S5
S 381
S 759
S 776
S 790
S 447
no NTD
MCE2730837
Rickettsiales bacterium 49624_E59 (lake water)
WP_011944657
Orientia tsutsugamushi str. Boryong
MBA2628750
Rickettsiaceae bacterium iso. MGR_bin156 (mineral soils, Mackay Glacier)
MBU6183802
MBU6184378
Rickettsiales bacterium str. REEB450 (rare earth elements -acid mine drainage)
S 1419
S 882
S 980
S 702
LepB-like
MBN8523153
MBN8523500
Rickettsiales bacterium str. UWPOB_RICK1 (activated sludge)
MBN8512064
MBN8512436
Rickettsiales bacterium str. UWPOB_RICK2 (activated sludge)
D
n = 385
bits
N
C
