# Supplementary material for: Metagenome diversity illuminates the origins of pathogen effectors
Source: mBio. 2024 Apr 2;15(5):e00759-23. doi: 10.1128/mbio.00759-23 (PMC11077975; doi:10.1128/mbio.00759-23)
Supplement: Fig. S6-S10 — Additional supplemental figures. [file mbio.00759-23-s0002.pdf]

FIGURE S6. Characteristics of *Rickettsia* Ankyrin Repeat 2 (RARP-2) proteins and related ankyrin repeat containing proteins.

(A) Comparison of the domain architecture for *Rickettsia* RARP-2 proteins (*R*. *typhi* str. Wilmington as an exemplar, NCBI acc. no. AAU04065) and the ANK repeat-containing protein DFA_04436 (XP_004360169) of the cellular slime mold *Cavenderia fasciculata* (Eumycetozoa; Dictyostelia; Acytosteliales). **(B)** Sequence logos (190) constructed to illustrate the conservation between ANK repeats within *R*. *typhi* RARP-2 and D DFA_04436 and between the two models. Repeats were manually stacked and visualized with AliView 1.28.1 (207). Amino acid coloring is described in the FIG. 3 legend. **(C)** Structure of the *R*. *typhi* RARP-2 C-terminal ANK repeat domain. **(*left*)** Alignment of the entire ANK repeat-containing domains between *R*. *typhi* RARP-2 and *C. fasciculata* DFA_04436. Alignment constructed using MUSCLE (189) (default parameters). Helices above ANK repeats are derived from the conserved ANK repeat structure (118). Red sequence illustrates the fourth ANK repeat present in RARP-2 of *R*. *typhi* str. TH1527 (AFE54444), *R*. *typhi* str. B9991CWPP (AFE55282) and the laboratory strain named "Wilmington" that differs from AAU04065 and remains unpublished (58). **(*right*)** Prediction of entire RARP-2 structure using Alphafold (197, 198). Additional ANK-like folds flanking the 11 ANK repeats predicted by comparative analyses are noted with question marks. **(D)** Summary of divergent RARP-2 (dRARP-2) proteins. N-terminal protease domains from each of the six sequences in the outer circle were used as a query in BlastP searches and determined to have greater similarity to a cohort (number in parentheses and size of dot) or itself only (Pyropec, or *Rickettsia* endosymbiont of *Pyrocoelia pectoralis*, a MAG from Davison *et al*. (73)) versus other

query sequences or any RARP-2 protein (center). All sequence information is provided in **Table S2**.

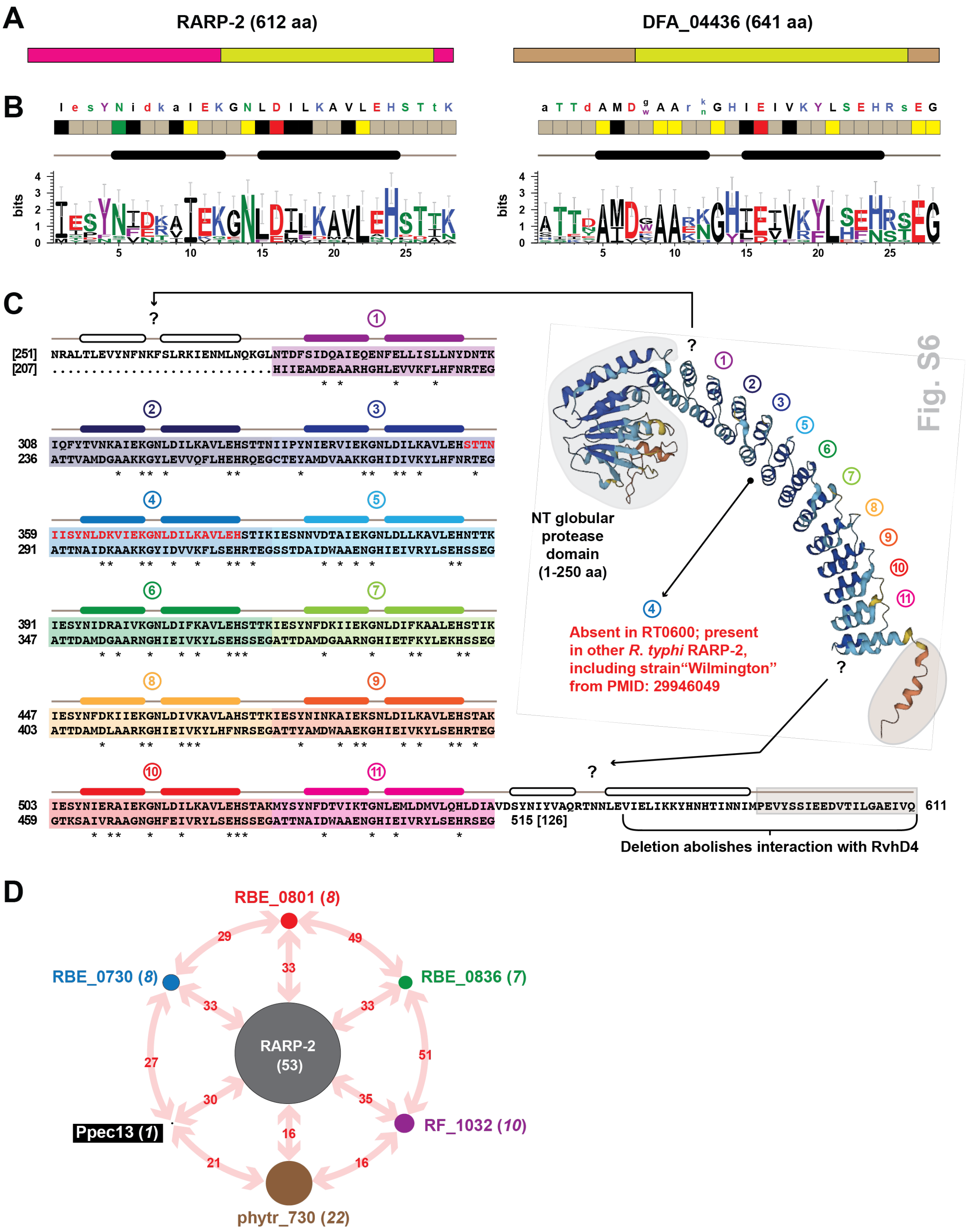
A
RARP-2 (612 aa)
DFA_04436 (641 aa)
B
bits
C
[251] NRALTLEVYNFNKFSLRKIENMLNQKGLNTDFSIDQAIEQENFELLISLLNYDNTK
[207] HIIEAMDEAARHGHLEVVKFLHFNRTEG
308 IQFYTVNKAIEKGNLDILKAVLEHSTTNIIPYNIERVIEKGNLDILKAVLEHSTTN
236 ATTVAMDGAAKKGYLEVVQFLHEHRQEGCTEYAMDVAAKKGHIDIVKYLHFNRTEG
359 IISYNLDKVIEKGNLDILKAVLEHSTIKIESNNVDTAIEKGNLDLLKAVLEHNTTK
291 ATTNAIDKAAKKGYIDVVKFLSEHRTEGSSTDAIDWAAENGHIEIVRYLSEHSSEG
391 IESYNIDRAIVKGNLDIFKAVLEHSTTKIESYNFDKIIEKGNLDIFKAALEHSTIK
347 ATTDAMDGAARNGHIEIVKYLSEHSSEGATTDAMDGAARNGHIETFKYLEKHSSEG
447 IESYNFDKIIEKGNLDIVKAVLAHSTTKIESYNINKAIEKSNLDILKAVLEHSTAK
403 ATTDAMDLAARKGHIEIVKYLHFNRSEGATTYAMDWAAEKGHIEIVKYLSEHRTEG
503 IESYNIERAIEKGNLDILKAVLEHSTAKMYSYNFDTVIKTGNLEMLDMVLQHLDIAVDSYNIYVAQRTNNLEVIELIKKYHNHTINNIMPEVYSSIEEDVTILGAEIVQ 611
459 GTKSAIVRAAGNGHFEIVRYLSEHSSEGATTNAIDWAAENGHIEIVRYLSEHRSEG 515 [126]
Deletion abolishes interaction with RvhD4
NT globular protease domain (1-250 aa)
Absent in RT0600; present in other R. typhi RARP-2, including strain "Wilmington" from PMID: 29946049
Fig. S6
D
RBE_0801 (8)
RBE_0730 (8)
RBE_0836 (7)
RARP-2 (53)
Ppec13 (1)
RF_1032 (10)
phytr_730 (22)

FIGURE S7. Phylogenomics analyses of candidate REMs (cREM-1-cREM-5). Protein information is provided in Table S2. All alignments done with MUSCLE (default parameters) (189) with conservation analyzed using WebLogo (190). Amino acid coloring is described in the FIG. 3 legend. Black boxes provide short names for MAGs from Davison *et al*. (73). **(A)** cREM-1 proteins are minimized in architecture relative to ancestral forms. Proteins with high similarity to RT0435 (cREM-1) are mostly conserved in *Rickettsia* genomes yet highly diverse (gray), sometimes duplicated and tandemly arrayed (red) and often components of larger modular proteins (cREM-1d). Inset illustrates cREM-1 similarity to tandem repeats in the scrub typhus effector OtDUB (CAM80065), which carries multiple eukaryotic-like domains (5, 124, 203) (described in FIG. 7). For brevity, alignment of a small conserved region shared across OtDUB repeat 1 (383-406), OtDUB repeat 2 (645-668), Blapp1 HJD67197, and Blapp1 HJD67198 is shown. Phylogeny estimation of 102 cREM-1 proteins indicates diversification of larger CREM-1 domain modular proteins and streamlining to a single cREM-1 protein in most *Rickettsia* genomes. Alignment was not masked (1544 total sites, 38.34% invariant). A maximum likelihood-based phylogeny was estimated with PhyML (185), using the Smart Model Selection (186) tool to determine the best substitution matrix and model for rates across aa sites (VT +G+F). Branch support was assessed with 1,000 pseudo-replications. Log likelihood of tree: -34728.6. **(B)** cREM-2 proteins diversified from an ancient gene duplication. Proteins with high similarity to RT0352 (cREM-a, red) and RT0351 (cREM-2b, light blue) are tandemly arrayed and mostly conserved in *Rickettsia* genomes, yet highly diverse from ancient forms (cREM-2d). Inset illustrates the conserved central region of cREM-2 proteins. Estimated phylogeny indicates cREM-2b

proteins are more divergent from cREM-2d proteins. Alignment was not masked (973 total sites, 43.4% invariant). Phylogeny estimated as described in panel **A** (final model LG +G). Branch support was assessed with 1,000 pseudo-replications. Log likelihood of tree: -25322.17. **(C)** cREM-3 proteins are highly conserved and present in other proteobacterial assemblies. These proteins are typically annotated as Pfam PF10877 (DUF2671: restricted to *Rickettsia* spp.). This sequence logo is for 107 non-redundant proteins obtained from searches against 'Rickettsiales', with proteins aligned using MUSCLE (default parameters). A more complete list of proteins is found in **Table S2**, though more sequences are likely retrievable using HMMER searches. At bottom, a pairwise alignment between *R. typhi* RT0206 and the most divergent subject retrieved from a BlastP search excluding 'Rickettsiales' is shown (hypothetical protein B7X02_01410 from Rhodospirillales bacterium 12-54-5, OYW13786.1). These proteins are 30% identical over the match shown. The residues noted with arrows in the sequence logo are shown over the pairwise alignment and below with a structure for *R. typhi* RT0206 predicted with Alphafold (197, 198). **(D)** cREM-4 proteins harbor a conserved pentapeptide repeat (PR). Schema shows alignment of 82 non-redundant cREM-4 proteins with illustration of the PR consensus sequence at top (208). A small conserved motif (inset) was also identified. **(E)** cREM-5 and cREM-5p have conserved central regions that lack similarity to proteins outside of *Rickettsia* and *Tisiphia* genomes. **(F)** One copy of cREM-5p from the RiClec (Endosymbiont of *Cimex lectularius*) genome is found on a RAGE transposon. General schema and annotation of RAGE genes follows previous reports (91, 92, 133).

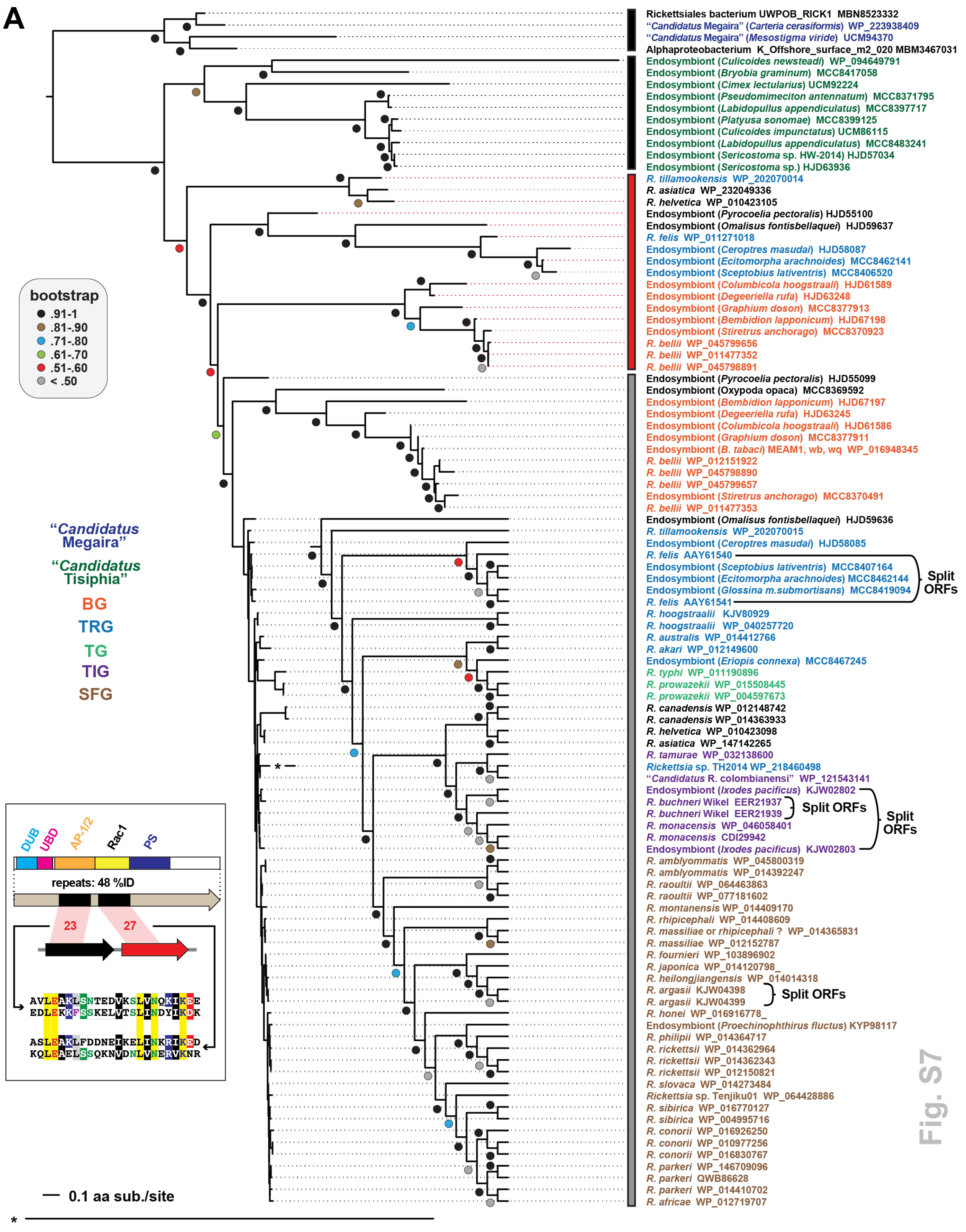

A
Rickettsiales bacterium UWPOB_RICK1 MBN8523332
"Candidatus Megaira" (Carteria cerasiformis) WP_223938409
"Candidatus Megaira" (Mesostigma viride) UCM94370
Alphaproteobacterium K_Offshore_surface_m2_020 MBM3467031
Endosymbiont (Culicoides newsteadi) WP_094649791
Endosymbiont (Bryobia graminum) MCC8417058
Endosymbiont (Cimex lectularius) UCM92224
Endosymbiont (Pseudomimeciton antennatum) MCC8371795
Endosymbiont (Labidopullus appendiculatus) MCC8397717
Endosymbiont (Platyusa sonomae) MCC8399125
Endosymbiont (Culicoides impunctatus) UCM86115
Endosymbiont (Labidopullus appendiculatus) MCC8483241
Endosymbiont (Sericostoma sp. HW-2014) HJD57034
Endosymbiont (Sericostoma sp.) HJD63936
R. tillamookensis WP_202070014
R. asiatica WP_232049336
R. helvetica WP_010423105
Endosymbiont (Pyrocoelia pectoralis) HJD55100
Endosymbiont (Omalisus fontisbellaquei) HJD59637
R. felis WP_011271018
Endosymbiont (Ceroptres masudai) HJD58087
Endosymbiont (Ecitomorpha arachnoides) MCC8462141
Endosymbiont (Sceptobius lativentris) MCC8406520
Endosymbiont (Columbicola hoogstraali) HJD61589
Endosymbiont (Degeeriella rufa) HJD63248
Endosymbiont (Graphium doson) MCC8377913
Endosymbiont (Bembidion lapponicum) HJD67198
Endosymbiont (Stiretrus anchorago) MCC8370923
R. bellii WP_045799656
R. bellii WP_011477352
R. bellii WP_045798891
Endosymbiont (Pyrocoelia pectoralis) HJD55099
Endosymbiont (Oxypoda opaca) MCC8369592
Endosymbiont (Bembidion lapponicum) HJD67197
Endosymbiont (Degeeriella rufa) HJD63245
Endosymbiont (Columbicola hoogstraali) HJD61586
Endosymbiont (Graphium doson) MCC8377911
Endosymbiont (B. tabaci) MEAM1, wb, wq WP_016948345
R. bellii WP_012151922
R. bellii WP_045798890
R. bellii WP_045799657
Endosymbiont (Stiretrus anchorago) MCC8370491
R. bellii WP_011477353
Endosymbiont (Omalisus fontisbellaquei) HJD59636
R. tillamookensis WP_202070015
Endosymbiont (Ceroptres masudai) HJD58085
R. felis AAY61540
Endosymbiont (Sceptobius lativentris) MCC8407164
Endosymbiont (Ecitomorpha arachnoides) MCC8462144
Endosymbiont (Glossina m.submortisans) MCC8419094
R. felis AAY61541
Split ORFs
R. hoogstraalii KJV80929
R. hoogstraalii WP_040257720
R. australis WP_014412766
R. akari WP_012149600
Endosymbiont (Eriopis connexa) MCC8467245
R. typhi WP_011190896
R. prowazekii WP_015508445
R. prowazekii WP_004597673
R. canadensis WP_012148742
R. canadensis WP_014363933
R. helvetica WP_010423098
R. asiatica WP_147142265
R. tamurae WP_032138600
Rickettsia sp. TH2014 WP_218460498
"Candidatus R. colombianensi" WP_121543141
Endosymbiont (Ixodes pacificus) KJW02802
R. buchneri Wikel EER21937
R. buchneri Wikel EER21939
Split ORFs
R. monacensis WP_046058401
R. monacensis CDI29942
Endosymbiont (Ixodes pacificus) KJW02803
Split ORFs
R. amblyommatis WP_045800319
R. amblyommatis WP_014392247
R. raoultii WP_064463863
R. raoultii WP_077181602
R. montanensis WP_014409170
R. rhipicephali WP_014408609
R. massiliae or rhipicephali ? WP_014365831
R. massiliae WP_012152787
R. fournieri WP_103896902
R. japonica WP_014120798
R. heilongjiangensis WP_014014318
R. argasii KJW04398
R. argasii KJW04399
Split ORFs
R. honei WP_016916778
Endosymbiont (Proechinophthirus fluctus) KYP98117
R. philipii WP_014364717
R. rickettsii WP_014362964
R. rickettsii WP_014362343
R. rickettsii WP_012150821
R. slovaca WP_014273484
Rickettsia sp. Tenjiku01 WP_064428886
R. sibirica WP_016770127
R. sibirica WP_004995716
R. conorii WP_016926250
R. conorii WP_010977256
R. conorii WP_016830767
R. parkeri WP_146709096
R. parkeri QWB86628
R. parkeri WP_014410702
R. africae WP_012719707
bootstrap
.91-1
.81-.90
.71-.80
.61-.70
.51-.60
< .50
"Candidatus Megaira"
"Candidatus Tisiphia"
BG
TRG
TG
TIG
SFG
DUB
UBD
AP-1/2
Rac1
PS
repeats: 48 %ID
23
27
AVLEAKLSNTEDVKSLVNQKIKEE
EDLEKKFSSKELVTSLINDYIKDK
ASLEAKLFDDNEIKELINKRIKED
KQLEAELSSQKNVDNLVNERVKNR
*
0.1 aa sub./site
*
Fig. S7

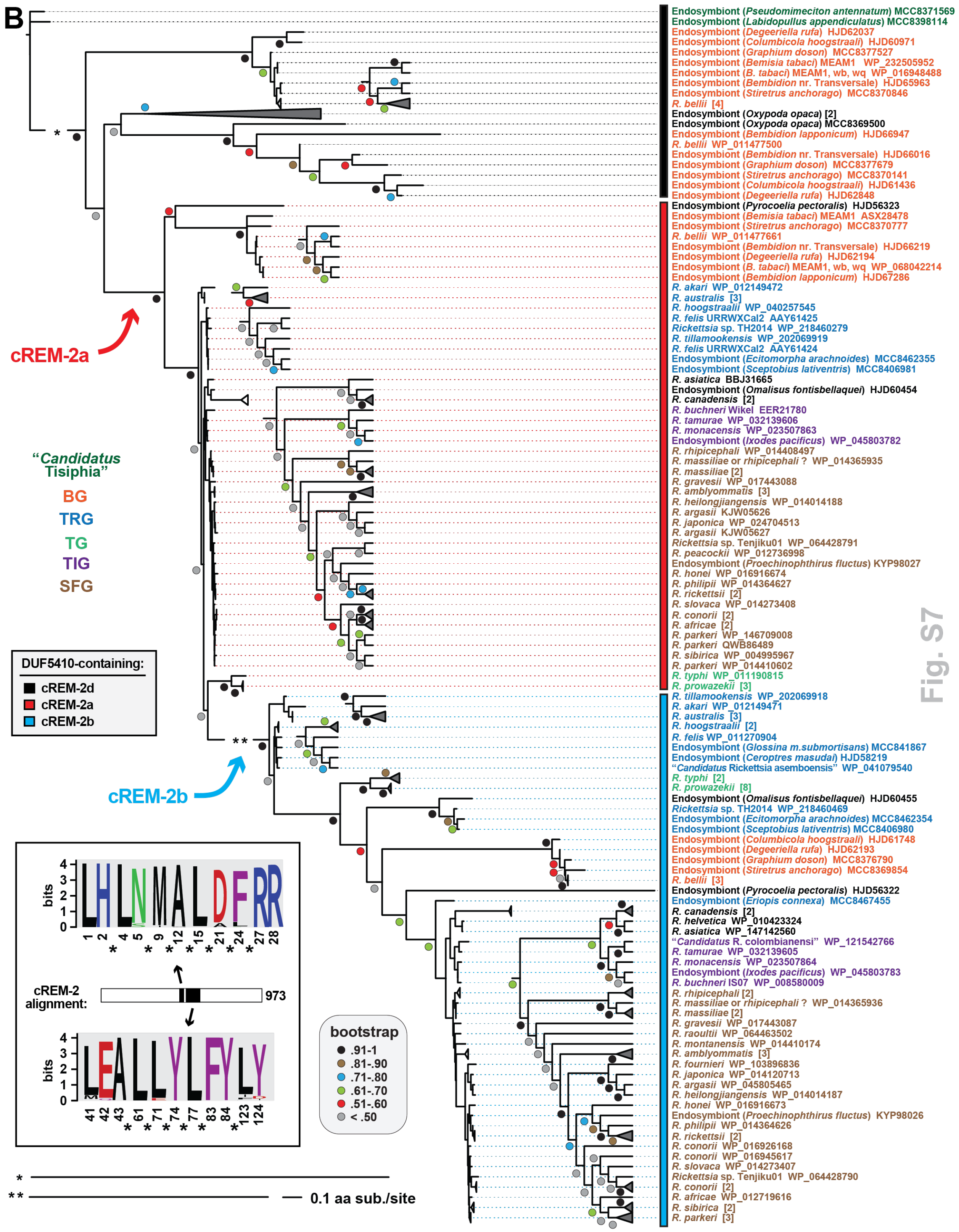

B
Endosymbiont (Pseudomimeciton antennatum) MCC8371569
Endosymbiont (Labidopullus appendiculatus) MCC8398114
Endosymbiont (Degeeriella rufa) HJD62037
Endosymbiont (Columbicola hoogstraali) HJD60971
Endosymbiont (Graphium doson) MCC8377527
Endosymbiont (Bemisia tabaci) MEAM1 WP_232505952
Endosymbiont (B. tabaci) MEAM1, wb, wq WP_016948488
Endosymbiont (Bembidion nr. Transversale) HJD65963
Endosymbiont (Stiretrus anchorago) MCC8370846
R. bellii [4]
Endosymbiont (Oxypoda opaca) [2]
Endosymbiont (Oxypoda opaca) MCC8369500
Endosymbiont (Bembidion lapponicum) HJD66947
R. bellii WP_011477500
Endosymbiont (Bembidion nr. Transversale) HJD66016
Endosymbiont (Graphium doson) MCC8377679
Endosymbiont (Stiretrus anchorago) MCC8370141
Endosymbiont (Columbicola hoogstraali) HJD61436
Endosymbiont (Degeeriella rufa) HJD62848
Endosymbiont (Pyrocoelia pectoralis) HJD56323
Endosymbiont (Bemisia tabaci) MEAM1 ASX28478
Endosymbiont (Stiretrus anchorago) MCC8370777
R. bellii WP_011477661
Endosymbiont (Bembidion nr. Transversale) HJD66219
Endosymbiont (Degeeriella rufa) HJD62194
Endosymbiont (B. tabaci) MEAM1, wb, wq WP_068042214
Endosymbiont (Bembidion lapponicum) HJD67286
R. akari WP_012149472
R. australis [3]
R. hoogstraalii WP_040257545
R. felis URRWXCal2 AAY61425
Rickettsia sp. TH2014 WP_218460279
R. tillamookensis WP_202069919
R. felis URRWXCal2 AAY61424
Endosymbiont (Ecitomorpha arachnoides) MCC8462355
Endosymbiont (Sceptobius lativentris) MCC8406981
R. asiatica BBJ31665
Endosymbiont (Omalisus fontisbellaquei) HJD60454
R. canadensis [2]
R. buchneri Wikel EER21780
R. tamurae WP_032139606
R. monacensis WP_023507863
Endosymbiont (Ixodes pacificus) WP_045803782
R. rhipicephali WP_014408497
R. massiliae or rhipicephali ? WP_014365935
R. massiliae [2]
R. gravesii WP_017443088
R. amblyommatis [3]
R. heilongjiangensis WP_014014188
R. argasii KJW05626
R. japonica WP_024704513
R. argasii KJW05627
Rickettsia sp. Tenjiku01 WP_064428791
R. peacockii WP_012736998
Endosymbiont (Proechinophthirus fluctus) KYP98027
R. honei WP_016916674
R. philipii WP_014364627
R. rickettsii [2]
R. slovaca WP_014273408
R. conorii [2]
R. africae [2]
R. parkeri WP_146709008
R. parkeri QWB86489
R. sibirica WP_004995967
R. parkeri WP_014410602
R. typhi WP_011190815
R. prowazekii [3]
R. tillamookensis WP_202069918
R. akari WP_012149471
R. australis [3]
R. hoogstraalii [2]
R. felis WP_011270904
Endosymbiont (Glossina m.submortisans) MCC841867
Endosymbiont (Ceroptres masudai) HJD58219
"Candidatus Rickettsia asemboensis" WP_041079540
R. typhi [2]
R. prowazekii [8]
Endosymbiont (Omalisus fontisbellaquei) HJD60455
Rickettsia sp. TH2014 WP_218460469
Endosymbiont (Ecitomorpha arachnoides) MCC8462354
Endosymbiont (Sceptobius lativentris) MCC8406980
Endosymbiont (Columbicola hoogstraali) HJD61748
Endosymbiont (Degeeriella rufa) HJD62193
Endosymbiont (Graphium doson) MCC8376790
Endosymbiont (Stiretrus anchorago) MCC8369854
R. bellii [3]
Endosymbiont (Pyrocoelia pectoralis) HJD56322
Endosymbiont (Eriopis connexa) MCC8467455
R. canadensis [2]
R. helvetica WP_010423324
R. asiatica WP_147142560
"Candidatus R. colombianensi" WP_121542766
R. tamurae WP_032139605
R. monacensis WP_023507864
Endosymbiont (Ixodes pacificus) WP_045803783
R. buchneri ISO7 WP_008580009
R. rhipicephali [2]
R. massiliae or rhipicephali ? WP_014365936
R. massiliae [2]
R. gravesii WP_017443087
R. raoultii WP_064463502
R. montanensis WP_014410174
R. amblyommatis [3]
R. fournieri WP_103896836
R. japonica WP_014120713
R. argasii WP_045805465
R. heilongjiangensis WP_014014187
R. honei WP_016916673
Endosymbiont (Proechinophthirus fluctus) KYP98026
R. philipii WP_014364626
R. rickettsii [2]
R. conorii WP_016926168
R. conorii WP_016945617
R. slovaca WP_014273407
Rickettsia sp. Tenjiku01 WP_064428790
R. conorii [2]
R. africae WP_012719616
R. sibirica [2]
R. parkeri [3]
cREM-2a
cREM-2b
"Candidatus Tisiphia"
BG
TRG
TG
TIG
SFG
DUF5410-containing:
cREM-2d
cREM-2a
cREM-2b
bits
cREM-2 alignment:
973
bootstrap
.91-1
.81-.90
.71-.80
.61-.70
.51-.60
< .50
0.1 aa sub./site
Fig. S7

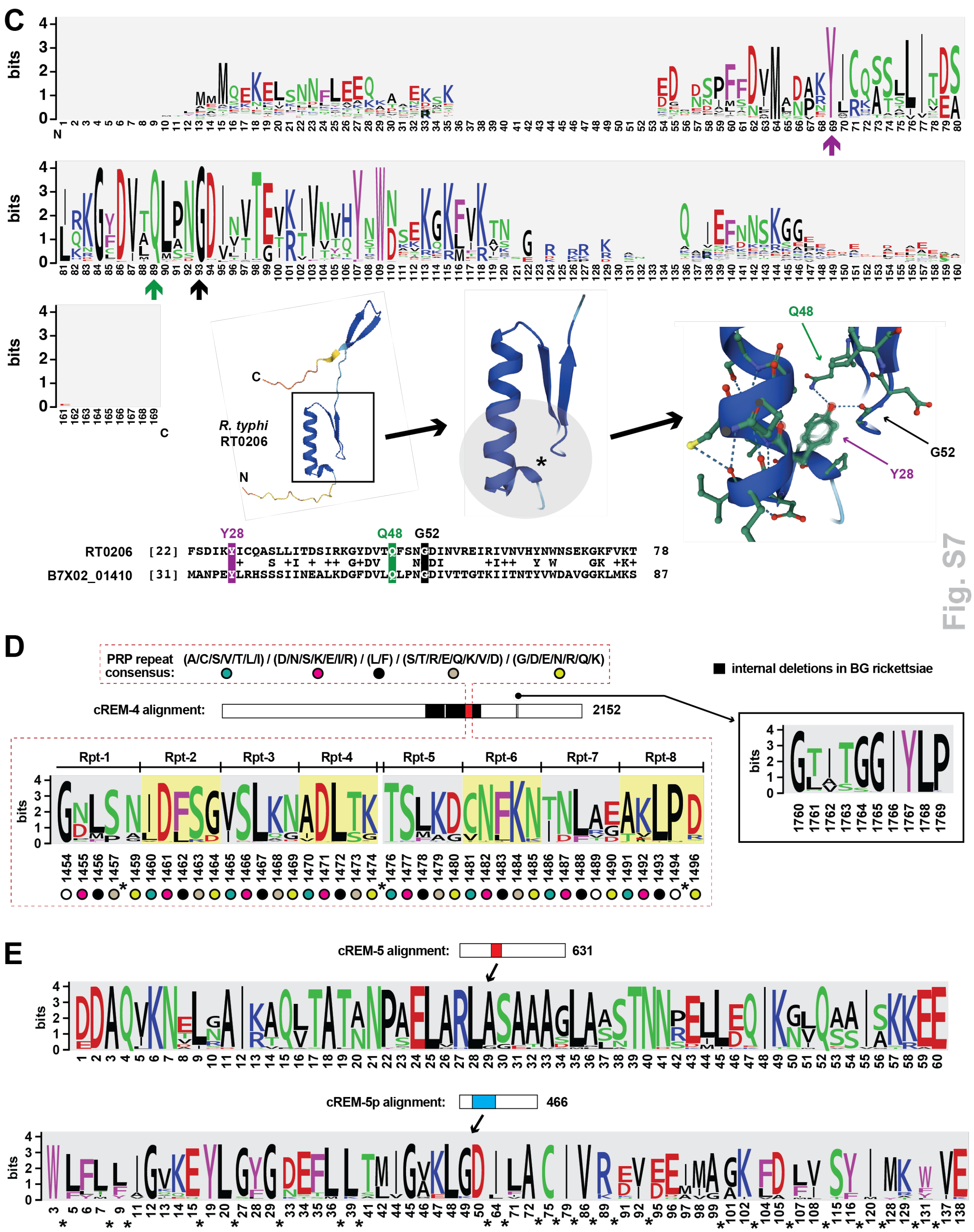
C
bits
R. typhi
RT0206
Q48
G52
Y28
RT0206 [22] FSDIKYICQASLLITDSIRKGYDVTQFSNGDINVREIRIVNVHYNWNSEKGKFVKT 78
+ S +I + ++ G+DV N DI +I++ Y W GK +K+
B7X02_01410 [31] MANPEYLRHSSSIINEALKDGFDVLQLPNGDIVTTGTKIITNTYVWDAVGGKLMKS 87
Fig. S7
D
PRP repeat consensus: (A/C/S/V/T/L/I) / (D/N/S/K/E/I/R) / (L/F) / (S/T/R/E/Q/K/V/D) / (G/D/E/N/R/Q/K)
internal deletions in BG rickettsiae
cREM-4 alignment:
2152
Rpt-1
Rpt-2
Rpt-3
Rpt-4
Rpt-5
Rpt-6
Rpt-7
Rpt-8
E
cREM-5 alignment:
631
cREM-5p alignment:
466

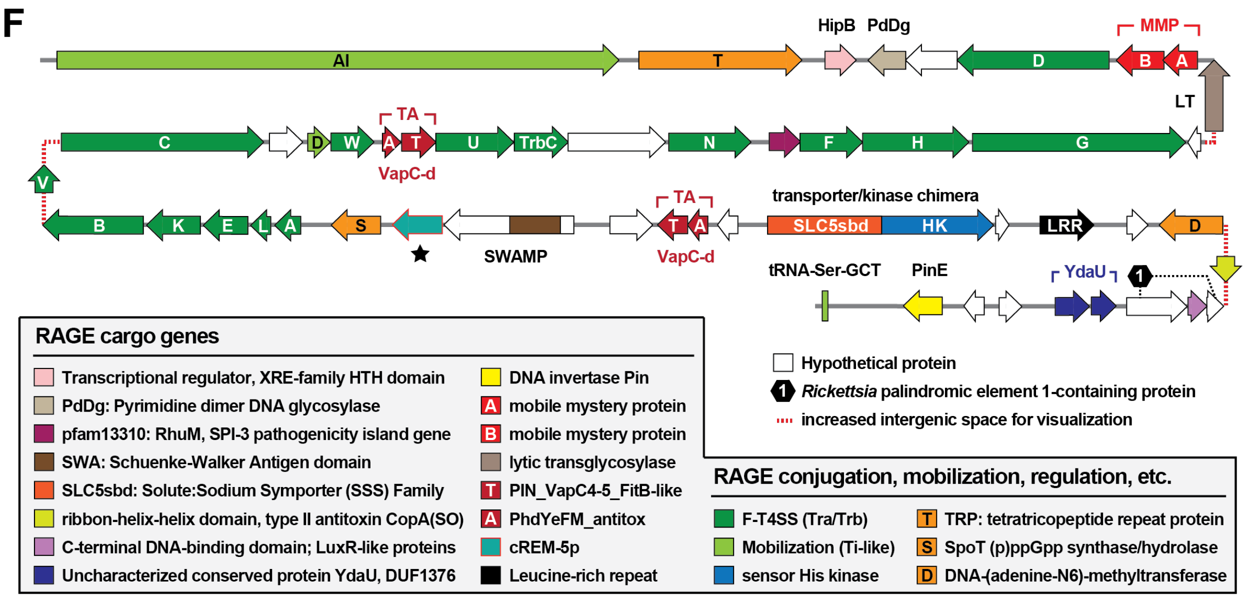
F
HipB
PdDg
MMP
AI
T
D
B
A
LT
TA
C
D
W
A
T
U
TrbC
N
F
H
G
VapC-d
V
transporter/kinase chimera
TA
B
K
E
L
A
S
SWAMP
T
A
SLC5sbd
HK
LRR
D
VapC-d
tRNA-Ser-GCT
PinE
YdaU
1
RAGE cargo genes
Transcriptional regulator, XRE-family HTH domain
PdDg: Pyrimidine dimer DNA glycosylase
pfam13310: RhuM, SPI-3 pathogenicity island gene
SWA: Schuenke-Walker Antigen domain
SLC5sbd: Solute:Sodium Symporter (SSS) Family
ribbon-helix-helix domain, type II antitoxin CopA(SO)
C-terminal DNA-binding domain; LuxR-like proteins
Uncharacterized conserved protein YdaU, DUF1376
DNA invertase Pin
A mobile mystery protein
B mobile mystery protein
lytic transglycosylase
T PIN_VapC4-5_FitB-like
A PhdYeFM_antitox
cREM-5p
Leucine-rich repeat
Hypothetical protein
1 Rickettsia palindromic element 1-containing protein
increased intergenic space for visualization
RAGE conjugation, mobilization, regulation, etc.
F-T4SS (Tra/Trb)
Mobilization (Ti-like)
sensor His kinase
T TRP: tetratricopeptide repeat protein
S SpoT (p)ppGpp synthase/hydrolase
D DNA-(adenine-N6)-methyltransferase

Fig. S7

FIGURE S8. Discovery of novel RickA architecture. Black boxes provide short names for 29 MAGs from Davison *et al*. (73). Amino acid coloring is described in the FIG. 3 legend. Sequence logos constructed with WebLogo 3 (190). Sequence information in Table S2. **(A)** General architecture of RickA proteins deduced from an alignment of 71 non-redundant sequences using MUSCLE (189) (default parameters). Consensus sequences for the WH2 (divided into two types based on one or two motifs) are shown at top, with divergent motifs illustrated. The remaining graphics illustrate the differences between *Tisiphia* and *Rickettsia* RickA C-terminal regions (summarized in the yellow ellipse). **(B)** Identification of a novel N-terminal repeat sequence in all RickA proteins. The repeat was identified by assessing within-protein BlastP matches, which revealed ~34 %ID between ~95 aa regions flanking the G-actin-binding domain (depicted in schema at top). Two examples are shown for a *Tishiphia* and *Rickettsia* repeat alignment, with secondary structure predictions indicating high conservation between repeats (199) . A comparison of the repeats R and R' all 71 non-redundant sequences is shown at bottom, with residues showing conservation in amino acid type (hydrophobicity, charge, aromaticity) shaded tan. This collectively illustrates that each RickA protein has greater within-repeat similarity than across-protein repeat similarity yet is constrained in overall amino acid type.

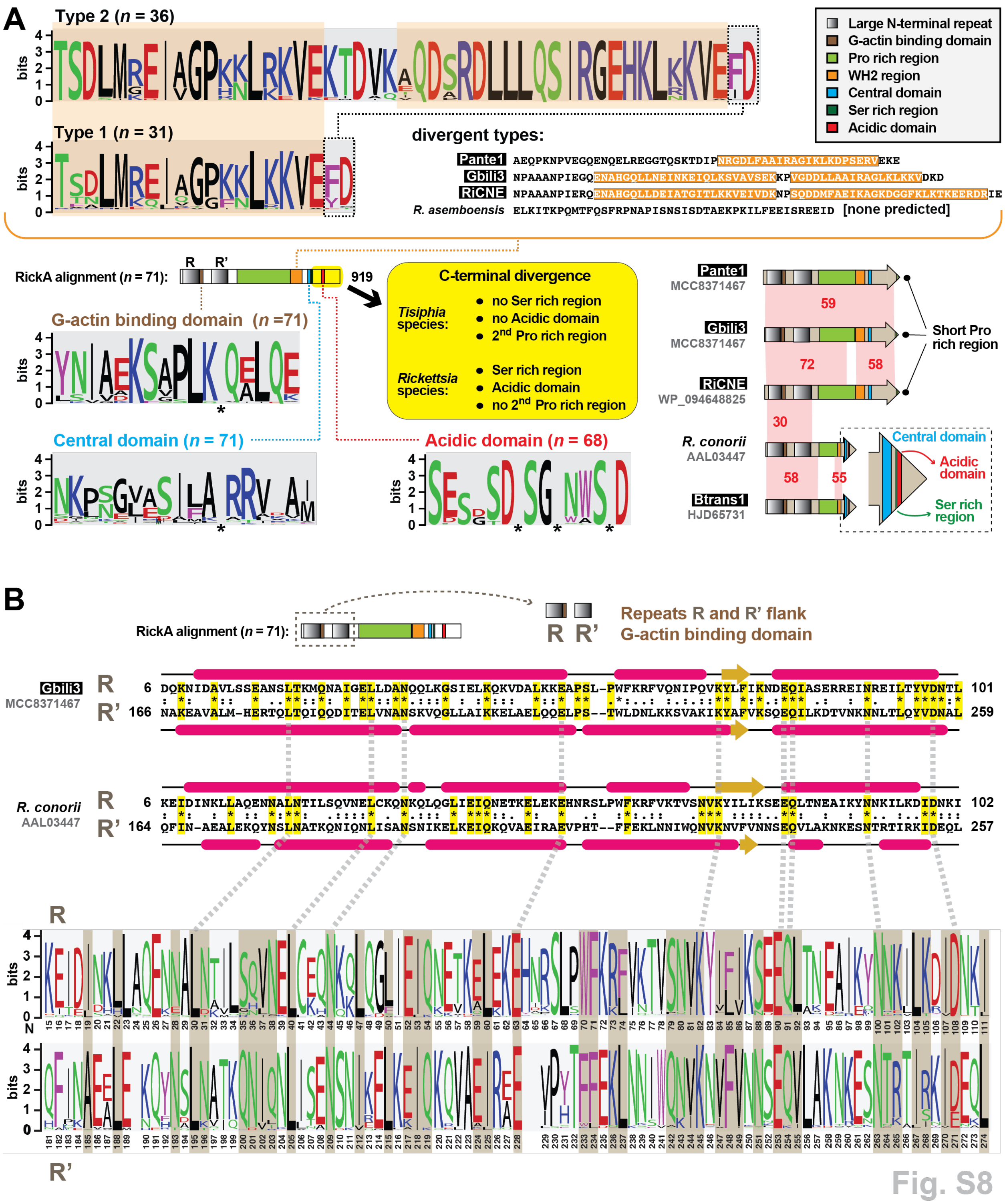
A
Type 2 (n = 36)
Type 1 (n = 31)
bits
Large N-terminal repeat
G-actin binding domain
Pro rich region
WH2 region
Central domain
Ser rich region
Acidic domain
divergent types:
Pante1
Gbili3
RiCNE
R. asembonensis
[none predicted]
RickA alignment (n = 71):
919
G-actin binding domain (n =71)
Central domain (n = 71)
Acidic domain (n = 68)
C-terminal divergence
Tisiphia species:
no Ser rich region
no Acidic domain
2nd Pro rich region
Rickettsia species:
Ser rich region
Acidic domain
no 2nd Pro rich region
MCC8371467
WP_094648825
R. conorii
AAL03447
Btrans1
HJD65731
Short Pro rich region
Central domain
Acidic domain
Ser rich region
59
72
58
30
58
55
B
Repeats R and R' flank
G-actin binding domain
R
R'
Gbili3
MCC8371467
R. conorii
AAL03447

Fig. S8

FIGURE S9. CDI-like/Rhs-like C-terminal toxin/antidote (CRCT/CRCA) modules occur in diverse rickettsial genomes. Black boxes provide short names for 29 MAGs from Davison *et al*. (73). Sequence information in **Table S2**. **(A)** Comparison of the *Cupriavidus taiwanensis* str. DSM 17343 CdiA/I module to several rickettsial large modular toxins, as well as select rCRCT/CRCA-3a modules. Domains were predicted with SMART (191). Amino acid similarity (%ID, red shading) was assessed using Blastp. **(B)** Structural analysis of a rCRCT/CRCA-3a module. (***top***) Alignment of *C. taiwanensis* CdiA with eight rickettsial rCRCT-3a toxins. Amino acid coloring is described in the **FIG. 3** legend. Structural information from *C. taiwanensis* CdiA (PDB:5T87) (209) is provided at top. Alignment performed using MUSCLE with default settings (189). (***bottom***) Modeling with Phyre2 (195) of six rCRCT-3a toxins to the CdiA-CT toxin structure of *C. taiwanensis* CdiA (PDB:5T87). All threading was done with >95% confidence. The *Jidaibacter* proteins were too divergent to model. Inset: while the *C. taiwanensis* module was solved as a co-complex (209), Phyre2 modeling could not thread rickettsial rCRCA-3a to the antidote CdiI within the TA co-complex. The best model (46.3% confidence, 12% ID) for *R. tamurae* rCRCA-3a was to the structure of *Drosophila melanogaster* MAST/Orbit N-terminal domain PDB:4G3A (210), indicating a similar helical bundle and similar topology as *C. taiwanensis* CdiI.

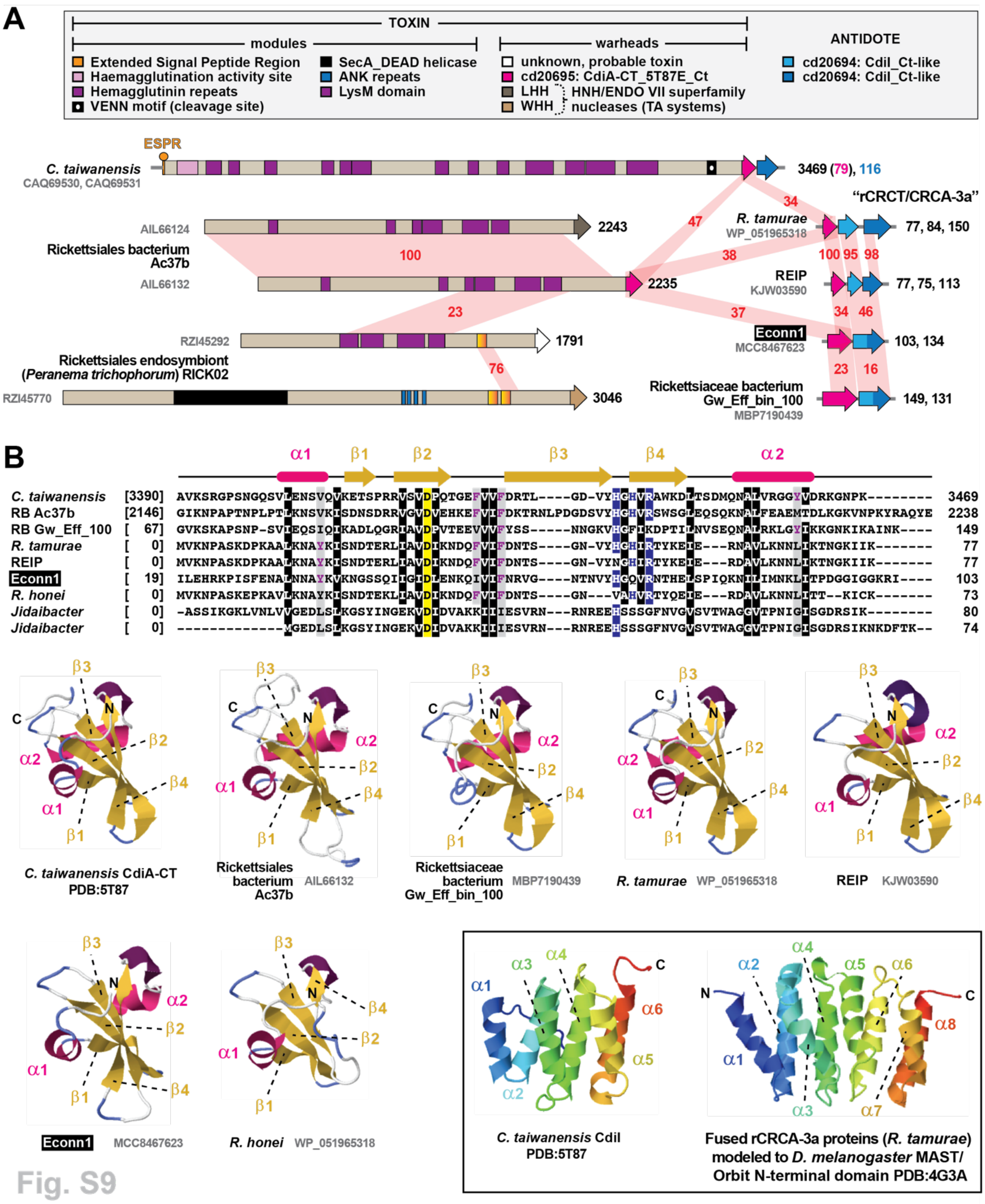
A
TOXIN
modules
Extended Signal Peptide Region
Haemagglutination activity site
Hemagglutinin repeats
VENN motif (cleavage site)
SecA_DEAD helicase
ANK repeats
LysM domain
warheads
unknown, probable toxin
cd20695: CdiA-CT_5T87E_Ct
LHH
WHH
HNH/ENDO VII superfamily nucleases (TA systems)
ANTIDOTE
cd20694: CdiI_Ct-like
cd20694: CdiI_Ct-like
ESPR
C. taiwanensis
CAQ69530, CAQ69531
3469 (79), 116
"rCRCT/CRCA-3a"
AIL66124
2243
Rickettsiales bacterium Ac37b
AIL66132
2235
RZI45292
1791
Rickettsiales endosymbiont (Peranema trichophorum) RICK02
RZI45770
3046
R. tamurae
WP_051965318
77, 84, 150
REIP
KJW03590
77, 75, 113
Econn1
MCC8467623
103, 134
Rickettsiaceae bacterium Gw_Eff_bin_100
MBP7190439
149, 131
B
C. taiwanensis [3390] AVKSRGPSNGQSVLENSVQVKETSPRRVSVDFQTGEFVVFDRTL----GD-VYHGHVRAWKDLTSDMQNALVRGGYVDRKGNPK-------- 3469
RB Ac37b [2146] GIKNPAPTNPLPTLKNSVKISDNSDRRVGVDVEHKEFVIFDKTRNLPDGDSVYHGHVRSWSGLEQSQKNALFEAEMTDLKGKVNPKYRAQYE 2238
RB Gw_Eff_100 [67] GVKSKAPSNP-SVIEQSIQIKADLQGRIAVDFVTEEVVVFYSS-----NNGKVHGFIKDPTILNVSEQNALRKLGYIKKGNKIKAINK---- 149
R. tamurae [0] MVKNPASKDPKAALKNAYKISNDTERLIAVDIKNDQFVIFDNTS----GN-VYHGHIRTYKEIE---RNAVLKNNLIKTNGKIIK------- 77
REIP [0] MVKNPASKDPKAALKNAYKISNDTERLIAVDIKNDQFVIFDNTS----GN-VYNGHIRTYKEIE---RDAVLKNNLIKTNGKIIK------- 77
Econn1 [19] ILEHRKPISFENALNNAYKVKNGSSQIIGIDLENKQIVIFNRVG-----NTNVYHGQVRNTHELSPIQKNILIMNKLITPDGGIGGKRI---- 103
R. honei [0] MVKNPASKEPKAVLKNAYKISNDTEKLIAVDIKNDQFVIFDNTS----GN---VAHVRTYQEIE---RDAVLKNNLITT--KICK------- 73
Jidaibacter [0] -ASSIKGKLVNLVVGEDLSLKGSYINGEKVDIDVAKKIIIESVRN---RNREEHSSSGFNVGVSVTWAGGVTPNIGISGDRSIK-------- 80
Jidaibacter [0] -------------VGEDLSLKGSYINGEKVDIDVAKKIIIESVRN---RNREEHSSSGFNVGVSVTWAGGVTPNIGISGDRSIKNKDFTK-- 74
C. taiwanensis CdiA-CT
PDB:5T87
Rickettsiales bacterium Ac37b AIL66132
Rickettsiaceae bacterium Gw_Eff_bin_100 MBP7190439
R. tamurae WP_051965318
REIP KJW03590
Econn1 MCC8467623
R. honei WP_051965318
C. taiwanensis CdiI
PDB:5T87
Fused rCRCA-3a proteins (R. tamurae) modeled to D. melanogaster MAST/Orbit N-terminal domain PDB:4G3A
Fig. S9

FIGURE S10. Identification of MAG proteins that have characteristics of reproductive toxins. Black boxes provide short names for 29 MAGs from Davison *et al*. (73). Sequence information in **Table S2**. [NOTE: The *Rickettsia* endosymbiont of *Oedothorax gibbosus* (NZ_OW370493) and *Rickettsia* endosymbiont of *Ceutorhynchus assimilis* (NZ_OU906081) assemblies were not included in our other analyses, as manuscripts supporting these assemblies were not published. We included them in this analysis due to their relevance to reproductive parasitism (RP) in *Rickettsia* species]. ***Top,*** architecture of the modular protein pLbAR_38, which is carried on plasmid pLbAR of *Rickettsia felis* str. LSU-Lb (gray inset) (92). Black triangles, proprotein convertase cleavage sites (211). We refer to this protein and its adjacent predicted antidote (not shown) as a CndA/B module (174), as the toxin encodes nuclease (CinB) and deubiquitinase (CidB) domains similar to RP toxins of certain wolbachiae that cause cytoplasmic incompatibility in arthropod hosts (168–171). We previously showed that pLbAR_38 shares similarity to a diverse assemblage of proteins from a narrow range of obligate intracellular bacteria, some of which are known reproductive parasites (5). Here, the remaining schema shows the result of a Blastp search against the NCBI nr database using pLbAR_38 (coordinates 1-3048, which excludes the ankyrin repeats) as the query (inset, color key for alignment scores). NOTE: each subject (numbered 1-24 at right) represents a distinct protein architecture, yet in some cases multiple similar proteins can be found for closely related species and strains). Yellow stars, novel RP toxins identified since our prior report (5). Matches for wolbachiae, *Cardinium* species (Bacteroidetes), *Diplorickettsia* species (*Gammaproteobacteria*), and *Rickettsiella* species (*Gammaproteobacteria*) are not shown to emphasize novel findings in

Rickettsiaceae. White numbers indicate % identity across significant alignments. Subjects missing internal sequence relative to pLbAR_38 (no. 4) are joined by dashed lines; subjects with large insertions relative to pLbAR_38 are adjusted accordingly (nos. 5, 14, and 15). Blurred-out regions within subjects depict sequences with no significant matches to pLbAR_38. Six proteins for the *Rickettsia* endosymbiont of *Ceutorhynchus assimilis* are boxed, with the arrow pointing to a seventh protein that is directly compared to a protein from the *Rickettsia* endosymbiont of *Adalia bipunctata* (unpublished assembly), which contains the ovarian tumor (OTU) cysteine protease (Pfam OTU, PF02338) domain also found in the Spaid male-killer toxin of *Spiroplasma poulsonii* sp. (172). Inset, alignment of the OTU protease domains of select bacteria: TEOG, *Tisiphia* endosymbiont of *Oedothorax gibbosus*; RECA, *Rickettsia* endosymbiont of *Ceutorhynchus assimilis*; REAB, *Rickettsia* endosymbiont of *Adalia bipunctata*. Amino acid coloring is described in the **FIG. 3** legend.

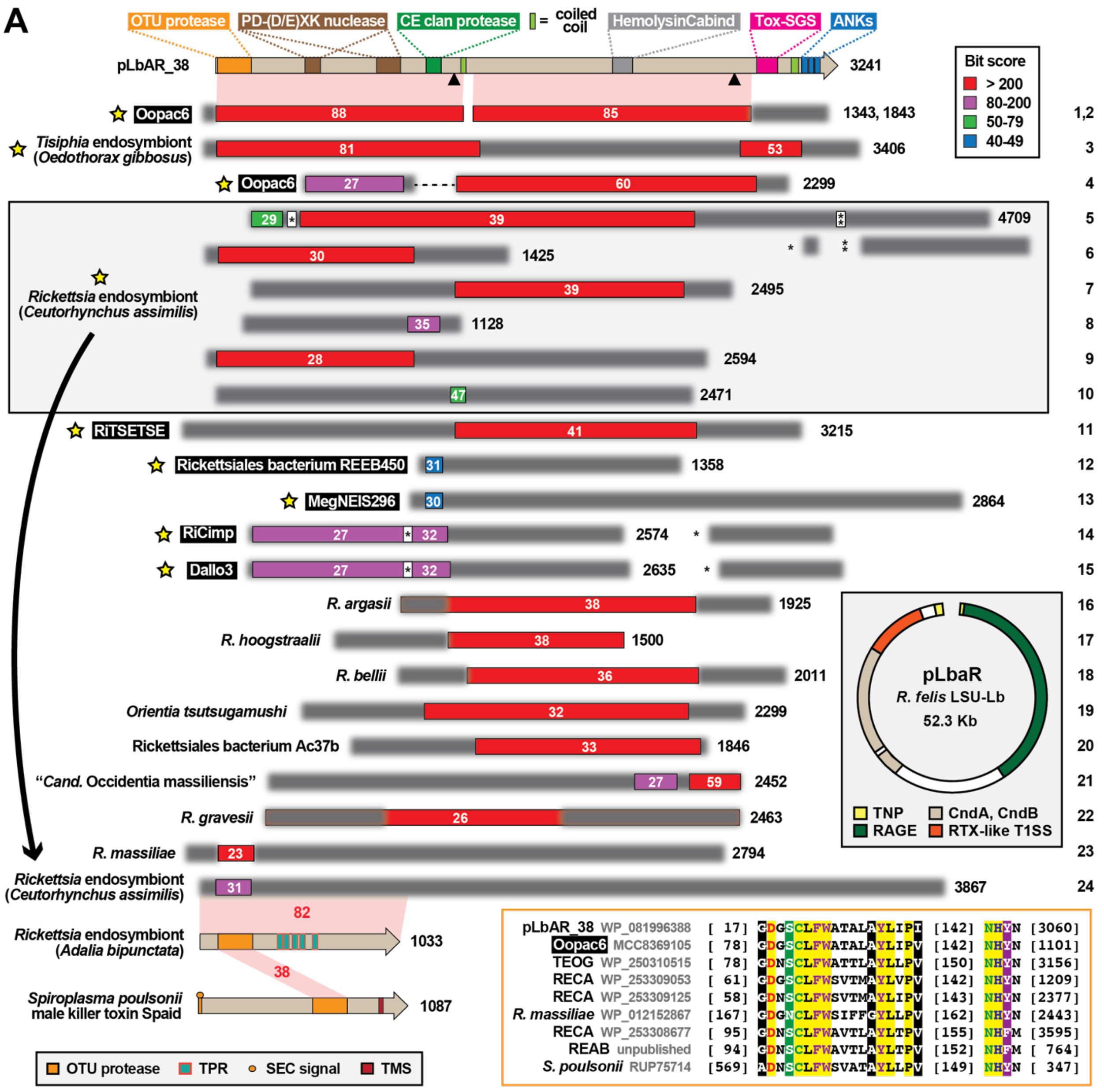
A
OTU protease
PD-(D/E)XK nuclease
CE clan protease
= coiled coil
HemolysinCabind
Tox-SGS
ANKs
pLbAR_38
3241
Bit score
> 200
80-200
50-79
40-49
Oopac6
1343, 1843
Tisiphia endosymbiont (Oedothorax gibbosus)
3406
2299
4709
1425
2495
1128
2594
2471
Rickettsia endosymbiont (Ceutorhynchus assimilis)
RiTSETSE
3215
Rickettsiales bacterium REEB450
1358
MegNEIS296
2864
RiCimp
2574
Dallo3
2635
R. argasii
1925
R. hoogstraalii
1500
R. bellii
2011
Orientia tsutsugamushi
2299
Rickettsiales bacterium Ac37b
1846
"Cand. Occidentia massiliensis"
2452
R. gravesii
2463
R. massiliae
2794
Rickettsia endosymbiont (Ceutorhynchus assimilis)
3867
pLbaR
R. felis LSU-Lb
52.3 Kb
TNP
CndA, CndB
RAGE
RTX-like T1SS
Rickettsia endosymbiont (Adalia bipunctata)
1033
Spiroplasma poulsonii male killer toxin Spaid
1087
OTU protease
TPR
SEC signal
TMS
pLbAR_38 WP_081996388
Oopac6 MCC8369105
TEOG WP_250310515
RECA WP_253309053
RECA WP_253309125
R. massiliae WP_012152867
RECA WP_253308677
REAB unpublished
S. poulsonii RUP75714

Fig. S10
